# *ΔNp73* isoform defines a *TP53*-mutant-like poor-risk subgroup of acute myeloid leukemia

**DOI:** 10.1016/j.xcrm.2025.102540

**Published:** 2026-01-08

**Authors:** Diego A. Pereira-Martins, Cesar Ortiz, Isabel Weinhäuser, Albertus T.J. Wierenga, Vincent van den Boom, Fatemeh Mojallali, Dominique Sternadt, Nisha K. van der Meer, Shanna M. Hogeling, Thiago M. Bianco, Prodromos Chatzikyriakou, Douglas R. Silveira, Emanuele Ammatuna, Antonio R. Lucena-Araujo, Lynn Quek, Gerwin Huls, Eduardo M. Rego, Jan Jacob Schuringa

**Affiliations:** 1 Department of Hematology, University Medical Center Groningen, University of Groningen, Groningen, the Netherlands; 2 Department of Medical Imaging, Haematology, and Oncology, Ribeirão Preto Medical School, University of São Paulo, Ribeirão Preto, SP, Brazil; 3 Center for Cell Based Therapy, São Paulo Research Foundation, Ribeirão Preto, SP, Brazil; 4 Hematology Division, LIM31, Faculdade de Medicina, University of São Paulo, São Paulo, Brazil; 5 Myeloid Leukaemia Genomics and Biology Group, School of Cancer and Pharmaceutical Sciences, King’s College London, London, UK; 6 Department of Genetics, Federal University of Pernambuco, Recife, Brazil

**Keywords:** acute myeloid leukemia, TP53-mutated AML, TP73, CEBPA, ferroptosis, poor prognosis prediction, AML PDX models, drug resistance, guanfacine, SREBP/SREBF

## Abstract

Among acute myeloid leukemia (AML) patients, a subgroup remains notoriously refractory to current treatment options, with underlying mechanisms poorly understood. Here, using a multi-omics approach, we reveal that this resistant patient subgroup is characterized by high expression of the oncogenic *TP73* isoform *ΔNp73*, exhibiting similarly poor outcomes as *TP53*-mutant AML. ΔNp73, which lacks a transcriptional activation domain but retains chromatin-binding properties, competes with *TP53* for specific gene targets, thereby downregulating TP53 signaling. We demonstrate that the transcription factor CEBPA controls *ΔNp73* expression in AML cells by binding to an intragenic enhancer region. Genetic or pharmacological inhibition of the transcriptional activity of CEBPA with guanfacine reduces *ΔNp73* levels and restores drug sensitivity involving ferroptosis-mediated cell death, acting synergistically with venetoclax. Our study sheds light on a previously undercharacterized poor-risk subgroup of AML, which may support patient stratification and inform treatment considerations.

## Highlights

• A subset of *TP53*wt patients displays similarities with *TP53*mut patients with poor prognosis
• This subset expresses high levels of *ΔNp73*, which lacks a transcriptional activation domain
• ΔNp73 retains chromatin-binding properties and interferes with TP53 signaling
• *ΔNp73* expression is controlled by CEBPA via binding to an intragenic enhancer

## Graphical abstract

**Figure:**
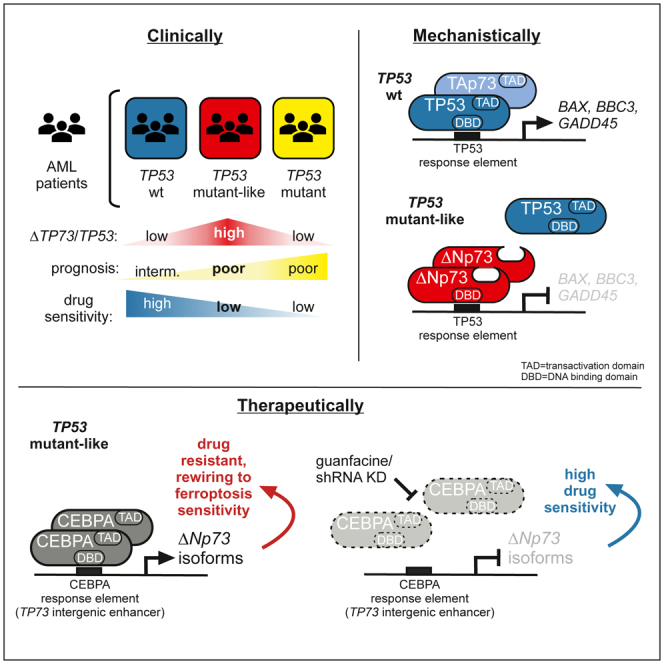

## Teaser

Pereira-Martins et al. identify an AML subgroup with poor prognosis marked by elevated ΔNp73, an oncogenic TP73 isoform*.* This isoform lacks a transcriptional activation domain and competes with TP53 for specific targets. ΔNp73 expression is regulated by CEBPA via binding to an intragenic enhancer, which is amendable to pharmacological targeting.

## Introduction

Acute myeloid leukemia (AML) with mutated *TP53* is recognized in the international consensus classification (ICC-2022) as a separate entity within the group of myeloid neoplasms with mutated *TP53*, including myelodysplastic syndrome (MDS) and MDS/AML with mutated *TP53.*^1^^,^^2^ *TP53* mutations are identified in roughly 10% of AML and MDS patients and are typically associated with complex cytogenetic abnormalities and a very poor outcome.^3^ Mutations within the *TP53* DNA-binding domain have been suggested to cause poor response to therapy-mediated cell death dependent on TP53 downstream signaling.^3^^,^^4^ *TP53*-mutated (*TP53*mut) clones that already exist prior the onset of full-blown AML can preferentially expand under genotoxic therapies due to selective pressure.^3^^,^^4^ Consequently, impaired induction of cell death contributes to the increased resistance of *TP53*mut AML/MDS blasts to both chemotherapy and venetoclax (VEN)-based treatments.^5^

While drug resistance is pronounced in *TP53*-mutated AML patients, dismal outcome due to the survival advantage of leukemic cells also occurs independently of *TP53* mutations.^6^ The *TP73* gene has been identified as a paralog of *TP53*, but the mutation rate in cancer is very low. They share three key domains with the p53 protein: the transactivation (TA) domain, the DNA-binding domain (DBD), and the oligomerization domain, with 29%, 63%, and 49% homology to TP53, respectively.^7^^,^^8^ Yet, in contrast to *TP53*, *TP73* can be transcribed into different isoforms using the extrinsic promoter 1 (P1) and the alternative intrinsic promoter P2 at the 5′ end to generate the carboxy-terminal spliced TA variant and the truncated delta N (ΔN) isoforms, which lack the TA domain.^9^ Both the TA and ΔN isoforms possess the DBD and the tetramerization domain, which allows them to oligomerize and bind to TP53/TP73 response elements.^7^ While the full-length TA isoform has been reported to activate the TP53 downstream signaling pathway, the ΔN isoform has been suggested to antagonize TP53 function.^10^ As such, knockout (KO) of TAp73 in mice enhanced the risk of tumor development and increased genomic instability, while ΔN-KO mice were more susceptible to DNA damage and p53-mediated apoptosis induction.^11^^,^^12^

Here, we show that a subgroup of AML patients, despite having a wild-type *TP53* (*TP53*wt), behaves as *TP53*mut. This occurs as a consequence of high expression of *ΔNp73*, which drives poor prognosis by inhibiting TP53 downstream signaling pathways causing resistance to drug-induced apoptosis. We identified that *ΔNp73* expression is regulated by an intragenic enhancer region in the *TP73* gene controlled by CEBPA, which can be targeted by the clinically graded compound guanfacine (GFC). Notably, GFC treatment of *TP53*wt/ΔNp73*-*high or *TP53*mut AML cells restored *TAp73* levels and induced ferroptosis-like cell death, representing a potential therapeutic approach for this AML subgroup with dismal prognosis.

## Results

### Identification of a *TP53*wt AML subgroup that shares similarities with *TP53*mut patients

**Figure 1:**
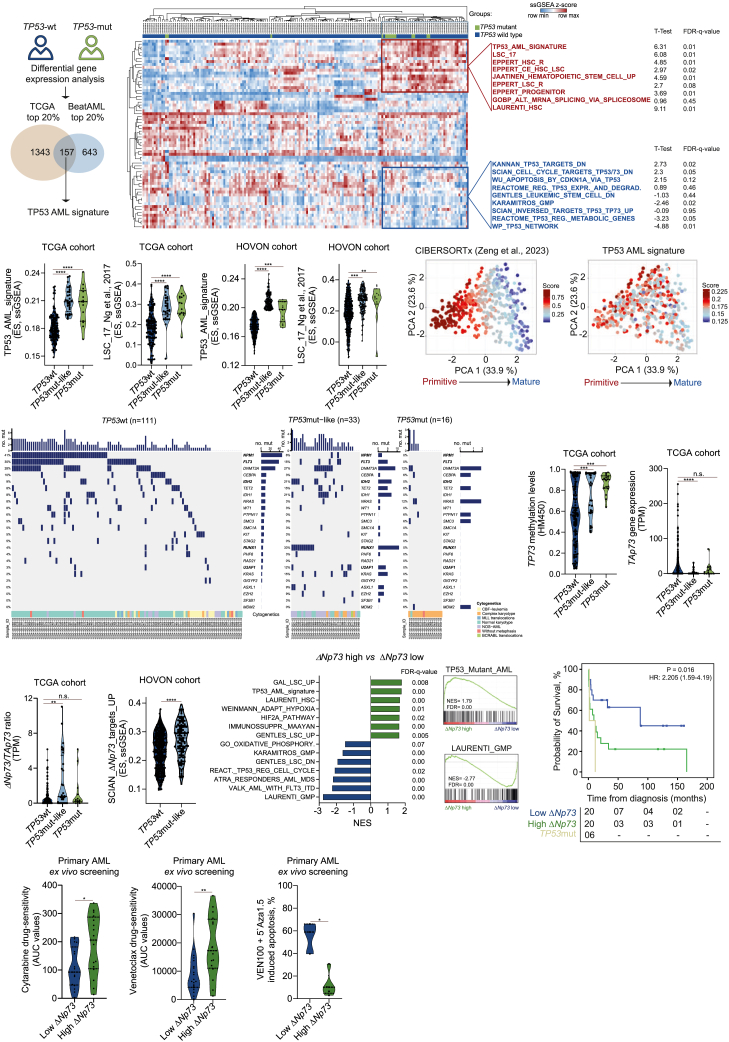
Enrichment analysis for genes associated with TP53 signaling identifies an AML subgroup with *TP53*mut-like (A) General workflow of the differential gene expression analysis comparing patient with *TP53*mut vs. *TP53*wt included in the TCGA cohort^13^ (*n* = 157) and BeatAML cohort^14^ (*n* = 447). The top 20% differentially expressed genes, upregulated in *TP53*mut AMLs from both datasets (157 genes), were used to create a *TP53* AML signature. (B) Heatmap depicting the ssGSEA projection of TCGA dataset for 173 AML samples on the collection of 65 gene sets associated with the TP53 signaling pathway and normal and malignant hematopoiesis (MSigDB v.7.1), defining a cluster of AML samples enriched for the TP53 AML signature. AML samples are annotated with the enrichment scores (ESs) for the ssGSEAs for each individual dataset. Data are clustered according to the hierarchical clustering for Spearman rank correlation. Top-scoring gene sets within the cluster with strong positive (in red) and negative (in blue) enrichment for the *TP53*mut signature are listed next to the heatmap, with their respective statistical analysis. (C) Violin plots displaying the ES for the TP53 AML signature and the LSC_17 signature^15^ for AML patients included in the TCGA cohort (*n* = 173) and HOVON (*n* = 530) cohort.^16^^,^^17^ Patients were categorized according to the *TP53* mutational status into *TP53*wt, *TP53*mut-like, and *TP53*mut. (D) Principal-component analysis (PCA) of 173 patients with AML from the TCGA cohort based on the composition of their cellular hierarchy.^18^ Right: the levels of TP53 AML signature per patient. (E) Oncoprint displaying the baseline mutations of the patients with *TP53*wt, *TP53*mut-like, and *TP53*mut AMLs in the TCGA cohort. Annotations regarding their cytogenetics are displayed at the bottom row. Genes in bold are the ones significantly different. (F–H) Violin plots displaying the methylation levels for *TP73* gene (F), the *TAp73* gene expression (G), and the ratio of expression between the *ΔNp73*/*TAp73* isoforms (H) for AML patients included in the TCGA cohort (*n* = 173). (I) Violin plot displaying the ES for the SCIAN_ΔNp73_targets_UP signature for AML patients included in the HOVON cohort. Patients were categorized according to the *TP53* mutational status into *TP53*wt and *TP53*mut-like (*n* = 517). (J) Gene Ontology (GO) and gene set enrichment analysis (GSEA) of *ΔNp73*^low^ and *ΔNp73*^high^ patients (*n* = 8) analyzed on the proteome of CD34^+^-sorted AML cells. NES, normalized enrichment score; FDR, false discovery rate. (K) The probability of overall survival (OS) in AML patients treated with 3 + 7-based protocols according to the *ΔNp73* levels (high versus low), compared to *TP53*mut patients. OS curves were estimated using the Kaplan-Meier method, and the log rank test was used for comparison. (L and M) Violin plots displaying the drug sensitivity to AraC (*n* = 33) and venetoclax (VEN, *n* = 36) (L) and the drug-induced apoptosis of VEN (100 nM) + 5-azacytidine (5′ Aza, 1.5 μM) (*n* = 8) (M) in *ex vivo*-treated primary AML samples (72 h). In (L), values are displayed as area under the curve (AUC), where high levels indicate resistance to therapy. Patients were dichotomized based on *ΔNp73* expression. The *p* values are indicated in the graphs; ∗*p* < 0.05; ∗∗*p* < 0.01; ∗∗∗*p* < 0.001; ANOVA and Bonferroni post-test.

To investigate the biological differences between *TP53*wt (without deletions of chromosome 17/17p) and *TP53*mut AML patients, we performed a differential gene expression analysis comparing *TP53*wt and mutated patients using transcriptome data of The Cancer Genome Atlas (TCGA)^13^ (*n* = 153, *TP53*mut: 14 patients, 9 with del17/17p) and BeatAML^14^ (*n* = 447, *TP53*mut: 31 patients, 10 with del17/17p) cohorts. *TP53* mutations included missense mutations (27 patients, 60%), splice mutations (9 patients, 20%), and truncating mutations (9 patients, 20%). Overall, 157 upregulated genes (considering the top 20%) were identified in *TP53*mut patients from both datasets. These genes were then collectively referred to as the *TP53* AML signature (Figure 1A). Next, single sample gene set enrichment analysis (ssGSEA) using 34,550 individual gene sets was performed on the TCGA cohort followed by unsupervised cluster analysis. A total of 65 gene sets (Table S1) associated with TP53 signaling and normal/malignant hematopoietic stem cell programs were differentially enriched in *TP53*mut patients. Upregulated terms in *TP53*mut patients included “Leukemic stem cell (LSC) up,” “Hematopoietic stem cell up,” and our developed “*TP53* AML signature,” while terms like “TP53 expression and degradation down,” “Apoptosis by CDKN1A,” and “TP73 targets” were downregulated (Figure 1B). Remarkably, a subset of *TP53*wt AML patients (comprising on average 22% of the *TP53*wt patients) also clustered together with *TP53*mut AMLs, indicating that these individuals share similar molecular programs (Figure 1B). Patients with *TP53*mut-like signatures displayed a higher frequency of complex karyotypes (33%) compared with *TP53*wt patients (4%) and no significant differences in the presence of del17/17p compared with *TP53*mut patients.

To further quantitatively investigate this *TP53*wt patient subgroup that exhibits molecular programs similar to *TP53*mut AMLs, we applied our *TP53* AML signature and calculated enrichment scores (ES) across the two cohorts and in an independent validation cohort of adult AML patients (HOVON cohort,^16^^,^^17^ *n* = 471). Increased enrichment for the TP53 AML signature was confirmed in *TP53*mut patients and also in a subgroup of *TP53*wt patients, which was subsequently categorized as *TP53* mutant-like (*TP53*mut-like) (Figures 1B and 1C; Table S2). Both *TP53*mut and *TP53*mut-like patients were also enriched for the LSC17^15^ signature, indicative for being a relatively immature AML subtype (Figure 1C). This observation was subsequently validated using CIBERSORTx estimation,^18^ which revealed that the TP53 AML signature was associated with primitive AMLs (Figure 1D). Overall survival (OS) analysis using TCGA and HOVON cohorts confirmed worse prognosis for *TP53*mut and *TP53*mut-like AMLs compared to patients with *TP53*wt (Figures S1A and S1B). It is important to note that *TP53*mut patients continued to exhibit significantly poorer OS compared to *TP53*mut-like AMLs (hazard ratio = 2.97, 95% confidence interval [CI]: 1.16–7.57, *p* = 0.022). Finally, mutational landscape analysis indicated that *FLT3-*ITD and *NPM1* mutations were more frequent in *TP53*wt patients, while *TP53*mut and *TP53*mut-like AMLs displayed a higher prevalence of *RUNX1*, *IDH2*, and spliceosome-related genes (Figure 1E). Mutations in genes related to the DNA-damage repair pathway (*PPM1D*, *MDM2*, *MDM4*, and *PHF6*) were not differentially present between the *TP53*wt and *TP53*mut or *TP53*mut-like.

### A high *ΔNp73*/*TAp73* ratio drives *TP53*mut-like phenotypes

To identify TP53 family members driving the *TP53*mut-like phenotype, we assessed the epigenome of *TP53*wt, *TP53*mut-like, and *TP53*mut patients using TCGA methylation array data (HM450^13^). Among the TP53 family of transcription factors, the *TP73* gene was highly methylated in *TP53*mut and *TP53*mut-like AMLs (Figure 1F). Two main groups of isoforms can be transcribed from the *TP73* locus: the transcriptionally active *TAp73* isoform, which is associated with the activation of TP53 downstream signaling, and truncated isoforms, collectively termed *ΔNp73*, whose function in AML has remained unknown (Figure S1C)*.* Consistent with increased DNA methylation, *TAp73* expression was reduced in *TP53*mut-like AML (Figure 1G). In contrast, we noted a strong upregulation of the truncated *ΔNp73* isoform relative to *TAp73* in *TP53*mut-like AMLs (Figure 1H). Expression of ΔNp73 in *TP53*mut-like AMLs was also confirmed at the protein level (Figure S1D). Given that *ΔNp73* retains its DNA-binding domain (Figure S1C), we hypothesized that the *ΔNp73/TP53* expression ratio might identify patients with TP53 pathway inhibition. Indeed, AML patients with a high *ΔNp73/TP53* ratio showed significantly worse OS than those with a low ratio, suggesting functional repression of TP53 signaling (Figure S1E). In line with these observations, *TP53*mut-like AML patients displayed increased expression of ΔNp73 targets (Figure 1I), while patients mutated for spliceosome-related genes (*U2AF1*, *SRSF2*, and *SF3B1*) displayed a high *ΔNp73/TAp73* ratio (Figures S1F–S1H).

Next, we evaluated *ΔNp73* expression using quantitative real-time PCR in a cohort of AML patients for whom we had previously generated label-free quantitative proteome data on CD34^+^/CD117^+^ cells.^19^^,^^20^^,^^21^ Proteomic analysis comparing *ΔNp73*^*high*^ vs. *ΔNp73*^*low*^ patients revealed enrichment for terms like “TP53 mutant AML,” “LSC up,” and “ΔNp73 targets up” in *ΔNp73*^*high*^ AMLs while terms like “L-GMP” and “oxidative phosphorylation” were downregulated (Figure 1J). Clinically, patients with *ΔNp73*^*high*^ had a very poor prognosis (Figure 1K), and *ex vivo* evaluation of primary AML samples revealed increased resistance to cytarabine (AraC), VEN, or VEN + azacitidine in CD34^+^ *ΔNp73*^*high*^ AML cells (Figures 1L and 1M). Altogether, these findings suggest that *ΔNp73* levels can be used as a marker to identify *TP53*mut-like AMLs.

### ΔNp73 outcompetes TP53 chromatin binding at target genes, thereby inhibiting TP53 downstream signaling

**Figure 2:**
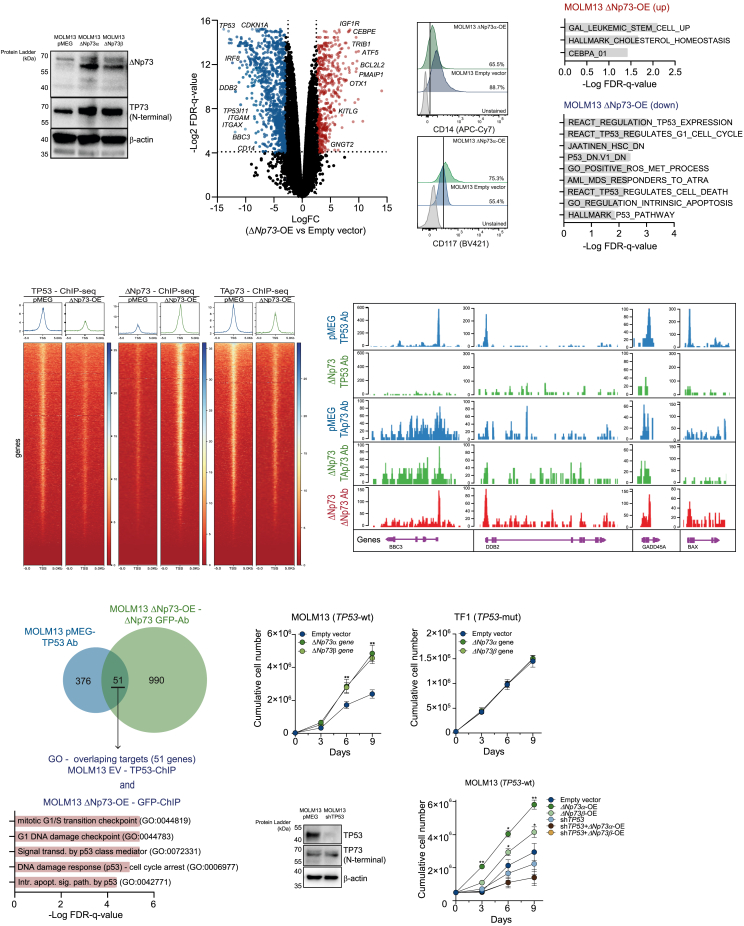
ΔNp73 overexpression is associated with downregulation of the TP53 signaling pathway in *TP53*wt AMLs (A) Western blot analysis for ΔNp73 and total TP73 in total cell extracts from MOLM13 cells transduced with lentivirus containing the EV (pMEG) or the ΔNp73α or ΔNp73β cDNA. (B) Volcano plot displaying the differentially expressed genes in MOLM13 cells with ΔNp73-OE versus EV control (*n* = 2). (C) Expression of CD14 and CD117 in MOLM13 EV (pMEG) and ΔNp73α-OE cells (*n* = 3). (D) GSEA analysis using the fold change values from the analysis depicted in (A). False discovery rate (FDR)-q values are indicated. (E) ChIP-seq data on MOLM13 cells used in (A) using antibodies against TP53 or GFP (for the GFP-ΔNp73 fusion), and TAp73. Heatmaps with signals ± 5 kb from the transcription start site (TSS) are shown. (F) Representative screenshots of TP53, TAp73, and ΔNp73 antibody binding at four TP53 target loci. (G) Venn diagram depicting overlapping peaks detected for the TP53 ChIP-seq in MOLM13 EV control cells and the GFP-ΔNp73 in MOLM13-ΔNp73 OE cells. Lower: GO analysis for the overlapping peaks (51 targets). (H and I) Cumulative cell count of MOLM13 (*TP53*wt, H) and TF1 (*TP53*mut, I) cells transduced with ΔNp73α, ΔNp73β, and EV control, cultured for 9 days (*n* = 4). (J) Western blot analysis for TP53 and total TP73 in total cell extracts from MOLM13 cells transduced with EV (pMEG) or the shRNA targeting the *TP53* gene (shTP53). Cumulative cell count of MOLM13 *TP53* KD cells (sh*TP53*) transduced with ΔNp73α, ΔNp73β, and EV control, cultured for 9 days, is shown in the right (*n* = 4). Data are reported as mean ± SEM for (H) and (I). The *p* values and cell lines are indicated in the graphs; ∗*p* < 0.05; ∗∗*p* < 0.01; ∗∗∗*p* < 0.001; ANOVA and Bonferroni post-test.

To explore the ΔNp73 downstream signaling pathway, we took advantage of the CCLE dataset for AML cell lines.^22^^,^^23^ These models exhibited significant heterogeneity in the *ΔNp73*/*TAp73* ratio (Figure S1I), with HL60 (which is functionally phenotypically *TP53*null due to low expression of the TP53 protein, but with full-length TAp73 protein levels being equally high as in other *TP53*wt cells, presumably exerting similar functions), U937 (with a *TP53* single-nucleotide variant of unknown significance), and NB4-R2 (*TP53* R248Q mutation) cells displaying the highest ratio of expression. Correlation analysis of the *ΔNp73*/*TAp73* ratio with gene expression programs across different cell lines indicated that a high *ΔNp73*/*TAp73* ratio co-existed with low expression of *TP53* target genes including *BAX*, *TP53* (p53), *TP73*, *CDKN1A* (p21), and *BID* (Figures S1J and S1K). We generated a MOLM13 ΔNp73-overexpression (OE) model (*TP53*wt, with a low baseline *ΔNp73*/*TAp73* ratio, Figure 2A) and performed transcriptome and chromatin immunoprecipitation sequencing (ChIP-seq) studies to identify direct transcriptional targets of ΔNp73 (OE GFP-tagged), endogenous TAp73, and endogenous TP53. ΔNp73-OE (Figure 2B) resulted in downregulation of classical TP53 target genes (*CDKN1A*, *TP53*, *BBC3*, and *DDB2*, Figure 2B) and genes associated with myeloid differentiation (*ITGAM*, *ITGAX*, and *CD14*) (Figures 2B and 2C). Conversely, genes associated with stemness, cholesterol metabolism, and drug resistance including *KITLG*, *BCL2L2*, *IGF1R*, and *CEBPE* were upregulated (Figure 2B). Gene set enrichment analysis revealed that MOLM13-ΔNp73 OE cells were enriched for terms like “LSC up,” “HALLMARK cholesterol homeostasis,” and “CEBPA 01,” while REACTOME processes like “regulation TP53 expression,” “TP53 regulates G1 cell cycle,” and “TP53 regulates cell death” were downregulated (Figure 2D), which was also observed in our analyses on *TP53*mut-like patients (Figures 1B and 1J).

ChIP-seq analysis of MOLM13-ΔNp73 OE cells revealed that ΔNp73 and TP53 compete for the same target genes. ΔNp73-OE resulted in a near-complete loss of p53 binding, with downregulation of known p53 target genes including *BBC3*, *DDB2*, *GADD45A*, and *BAX*, but also of *TP53* itself (Figures 2E and 2F). Overlapping TP53 binding sites in control cells with ΔNp73 binding sites in ΔNp73-OE cells confirmed ΔNp73 blockage of the TP53 signaling pathway, with genes identified in both conditions (*n* = 51) being related to processes like “Signaling transduction by p53 class mediator,” “DNA damage response (p53),” and “Intrinsic apoptosis signaling pathway by p53” (Figure 2G).

### ΔNp73-OE drives cellular proliferation in *TP53*wt AML cells and engraftment of primary APL cells in NSGS mice

To further characterize the molecular consequences of high *ΔNp73* levels in AML, we performed lentiviral OE of *ΔNp73α* and *ΔNp73β* isoforms in a panel of AML cell lines (*TP53*wt: *n* = 5, *TP53*mut: *n* = 7). *ΔNp73*-OE levels ranged from 4.29- to 23.85-fold when compared to their empty vector (EV) control, reaching similar levels as observed in primary AML samples within the *ΔNp73*^high^ group included in Figures 1J–1N. ΔNp73-OE was able to significantly enhance proliferation in *TP53*wt cell lines (data for MOLM13 are shown in Figure 2H), while no difference was observed for TF1 *TP53*mut cells (Figure 2I). ΔNp73-OE in MOLM13 cells, where the *TP53* gene was downregulated using a lentiviral short hairpin RNA (shRNA) approach, did not enhance proliferation (Figure 2J). Likewise, ΔNp73-OE enhanced cell proliferation of primary *TP53*wt AML blasts as well as of an acute promyelocytic leukemia (APL) patient, albeit with heterogeneity in the extent to which AMLs benefited from either the ΔNp73β or ΔNp73α isoform (Figure S1L).

ΔNp73α and ΔNp73β were also overexpressed in cord blood-derived CD34^+^ cells to study effects on normal human hematopoietic stem/progenitor cells. A significant increase in cellular growth over a period of 5 weeks was observed upon ΔNp73-OE, whereby a higher percentage of CD34^+^ cells was maintained at week 5 and colony formation capacity was enhanced at week 5 (Figures S1M and S1N), with no changes in differentiation across the conditions (data not shown).

Next, we assessed the potential of ΔNp73α-OE to enhance engraftment of primary patient samples *in vivo* in patient-derived xenograft (PDX) models. Primary APL cells were chosen as a model for *PML-RARα*-driven leukemia as they are known to be notoriously difficult to engraft (Figure S2A). Following transplantation in NSGS mice, APL-ΔNp73α-OE cells showed improved engraftment in peripheral blood at day 70 (Figure S2B). Moreover, APL-ΔNp73α-OE preserved a more immature phenotype defined by CD117 expression (often lost in PDX models for APL) and a more blast-like morphology characterized by a high nuclear:cytoplasm ratio, visible nucleoli, and the presence of Auer rods (Figures S2C–S2E). Additionally, spleen weight and spleen engraftment of GFP^+^huCD45^+^huCD33^+^ APL cells were notably higher in APL-ΔNp73α-OE mice compared to EV control mice (Figures S2F–S2H). To test whether the engrafted ΔNp73α-OE cells displayed a more aggressive phenotype, we sorted APL blasts (huCD45^+^huCD117^+^huCD33^+^) from ΔNp73α-OE/control mice and performed an *ex vivo* drug screening with *all-trans* retinoic acid (ATRA) and arsenic trioxide (ATO). Our results showed that APL-ΔNp73α-OE cells were more resistant to ATRA and ATO therapy compared to EV controls (Figure S2I). To further confirm our findings, we generated three independent AML PDX models (with heterogeneous genetic backgrounds but *TP53*wt) by transplanting MISTRG mice with AML blasts transduced with EV or ΔNp73α-OE. As observed for the APL models, ΔNp73α-OE was associated with superior engraftment in the bone marrow and with increased colonization of distal organs such as spleen and liver (Figures S2J–S2L).

### ΔNp73-OE imposes drug resistance in *TP53*wt AML

**Figure 3:**
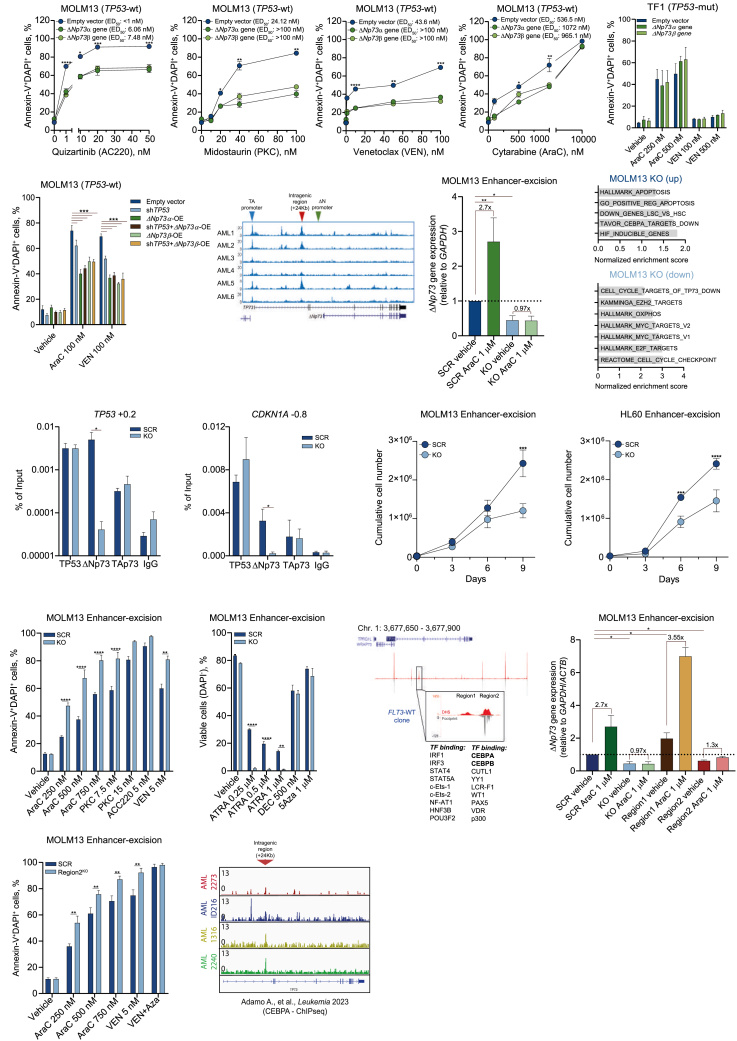
ΔNp73 expression is associated with drug resistance and is regulated by an intragenic region in the TP73 gene (A) MOLM13 cells (ΔNp73-OE and EV control) were treated with FLT3 inhibitors quizartinib (AC220) and midostaurin (PKC) and AML-related drugs venetoclax (VEN) and cytarabine (AraC) for 72 h. Apoptosis and viable cell numbers were assessed by flow cytometry. Experiments were performed in quadruplicates. Results are expressed as the mean ± standard error of the mean (SEM). ED_50_, half maximal effective concentration (*n* = 4). (B and C) Drug-induced apoptosis in TF1 cells (ΔNp73-OE and EV control) (B) and MOLM13 cells (transduced with shTP53 and ΔNp73-OE, as depicted in the figure) (C) treated with AML-related drugs (AraC and VEN; concentrations indicated in the plots, 72 h) detected by flow cytometry (*n* = 4). (D) DNAseI gene tracks in six AML samples from the BLUEPRINT consortium. The red arrows denote highly accessible sites (+24 kb from the TSS) in the *TP73* gene. The blue arrow denotes the TA promoter, and the green arrow denotes the ΔN promoter of the *TP73* gene locus. (E) Relative mRNA expression levels of Δ*Np73* after Cas9-mediated *TP73* enhancer excision in MOLM13 cells (MOLM13-KO) at baseline and upon AraC treatment (1 μM, 48 h) (*n* = 4). (F) GSEA analysis using the fold change values from the RNA-seq analysis comparing MOLM13-KO versus MOLM13-SCR cells (*n* = 2). (G) *TP53* (+0.2) and *CDKN1A* (−0.8) ChIP-qPCRs with error bars representing SEM based on three independent experiments. (H) Cumulative cell count of Cas9-mediated excision of TP73 intragenic enhancer region in MOLM13 and HL60 cells (KO versus SCR control) cultured for 9 days (*n* = 4). (I and J) Drug-induced apoptosis (I) and viable cell counts (J) in MOLM13-KO cells treated with AML-related drugs (drugs and concentrations indicated in the plots, 72 h) detected by flow cytometry (*n* = 4). (K) Genome browser screenshots of DNA hypersensitivity sites (DHSs) and digital footprints of the TP73 intragenic enhancer region in the *TP73* loci, revealing the two regions of the intragenic enhancer. Results from motif analysis are displayed at the bottom. (L) Relative mRNA expression levels of Δ*Np73* after Cas9-mediated *TP73* enhancer excision of the separate regions 1 and 2 in MOLM13 cells (MOLM13-KO included as a control) at baseline and upon AraC treatment (1 μM, 48 h) (*n* = 4). (M) Drug-induced apoptosis in region 2 KO MOLM13 cells treated with AML-related drugs (drugs and concentrations indicated in the plots, 72 h) detected by flow cytometry (*n* = 4). (N) Representative screenshots of CEBPA antibody binding at the TP73 enhancer region in primary AML samples.^24^ Data are reported as mean ± SEM for (A)–(C), (E), (H)–(J), (L), and (M). The *p* values and cell lines are indicated in the graphs; ∗*p* < 0.05; ∗∗*p* < 0.01; ∗∗∗*p* < 0.001; ANOVA and Bonferroni post-test.

Given that ΔNp73 blocked TP53 chromatin binding and downstream signaling essential for apoptosis induction, we tested several AML drugs in our panel of cell lines upon ΔNp73-OE. Overexpression of ΔNp73 in MOLM13 and MV4-11 cells (both carrying *FLT3*-ITD mutations) resulted in resistance to treatment with FLT3-ITD inhibitors (midostaurin [PKC] and quizartinib [AC220]), VEN, and AraC in comparison with the EV control (Figures 3A and S3A). Similar results were also observed for HL60 cells (*TP53* null, with *TAp73*wt functions), where ΔNp73-OE resulted in increased resistance to VEN and low dose of AraC (Figure S3B).

In *TP53*mut AMLs, ΔNp73-OE did not affect drug-induced apoptosis in TF1 cells (*TP53* I251fs) (Figure 3B). In KG1 cells (*TP53* c.672 + 1G>A – protein loss of function),^25^ ΔNp73-OE was associated with increased sensitivity to VEN (Figure S3C). To investigate whether ΔNp73-OE operates redundantly in the absence of functional TP53, typically the consequence of *TP53* mutations, we assessed drug resistance to AraC and VEN both with and without ΔNp73-OE in our MOLM13 TP53-knockdown (KD) model (Figure 2J). In the MOLM13 control (EV) cells, TP53-KD significantly increased drug resistance, irrespective of the drug administered (Figure 3C). Similarly, ΔNp73-OE also markedly increased drug resistance, but this was not further enhanced upon TP53-KD, suggesting that ΔNp73 imposes drug resistance by downregulation of the TP53 signaling pathway.

### Expression of ΔNp73 is regulated by an intragenic enhancer region

We next aimed to understand the underlying mechanism regulating *ΔNp73* expression in AML cells. Analysis of chromatin accessibility data (DNAseI-seq) retrieved from the BLUEPRINT consortium (no. 282510, BLUEPRINT) revealed no significant differences in the accessibility of the primary *TP73* promoter region (associated with *TAp73* expression) or the second promoter (associated with *ΔNp73* expression) across AML samples. However, we observed high heterogeneity in chromatin accessibility within an intragenic region located 24 kb downstream of the transcription start site, which was previously identified in adult T cell leukemia^26^ (Figure 3D). In BLUEPRINT AML samples (*n* = 16), both total *TP73* and *ΔNp73* expression were positively correlated with chromatin accessibility at its intragenic region (*TP73* rho Pearson: 0.66, 95% CI: 0.25 to 0.87, *p* = 0.0047; *ΔNp73* rho Pearson: 0.54, 95% CI: 0.08 to 0.81, *p* = 0.025). To investigate whether this enhancer would drive *ΔNp73* expression, CRISPR-Cas9 was used to delete this region (3,042 bp in length) in MOLM13 cells (enhancer-KO cells). In the enhancer-KO cells the baseline *ΔNp73* expression in untreated cells was significantly reduced, in line with the global changes in the transcriptional program associated with upregulation of processes like “GO_positive_regulation_of_apoptosis,” “TP73_targets_up,” and “LSC_down” (Figures 3E and 3F). Expression levels of neighboring genes *WRAP73* and *TPRG1L* remained stable upon deletion of the enhancer, suggesting no destabilization of the surrounding region (Figures S3D and S3E). TP53/TP73 family members are typically upregulated during stress conditions, and while treatment with high-dose AraC indeed resulted in a 2.7-fold upregulation of *ΔNp73* in MOLM13 scrambled (SCR) control cells, this was completely abrogated in enhancer-KO cells, indicating that this region directly controls *ΔNp73* expression under stress conditions (Figure 3E). ChIP-qPCR analysis revealed a significant reduction in binding of ΔNp73 to TP53 regulatory elements, with no significant changes in TAp73 or TP53 binding in enhancer-KO cells (Figure 3G). Functionally, deletion of the enhancer in a *TP53*wt cell model (MOLM13) and a *TAp73*-dependent model (HL60 cells, TP53 null phenotype) reduced AML cell proliferation and increased sensitivity to drug-induced apoptosis with several chemotherapeutic agents used in AML treatment, except for hypomethylating agents (HMAs) (Figures 3I, 3J, S3F, and S3G).

### CEBPA drives ΔNp73 expression, which can be targeted by GFC

Next, we aimed to unravel which transcription factors would potentially bind to the enhancer region, thereby controlling *ΔNp73* expression. Using our previously published digital footprinting data on AML subclones,^20^^,^^27^^,^^28^ we performed an in-depth analysis of the enhancer region, revealing the presence of two subregions (Figure 3K). To investigate which subregion specifically regulates *ΔNp73* expression, we performed CRISPR-KO of each region individually. While removal of region 1 did not affect *ΔNp73* expression in MOLM13 cells, region 2 deletion (999 bp in length) resulted in similar results as observed in the complete enhancer-KO cells, suggesting *ΔNp73* expression to be regulated by region 2 (Figure 3L), and increased AraC- and VEN-induced apoptosis (Figure 3M). Again, no destabilization of neighboring genes was observed (Figure S3H). Region 2 contained response elements for several transcription factors, including binding sites for CEBPA (Figure 3K). ChIP-seq analysis identified direct CEBPA binding to the intragenic regulatory region of the *TP73* locus, supporting its role in modulating *TP73* isoform expression in AML (Figure 3N).

**Figure 4:**
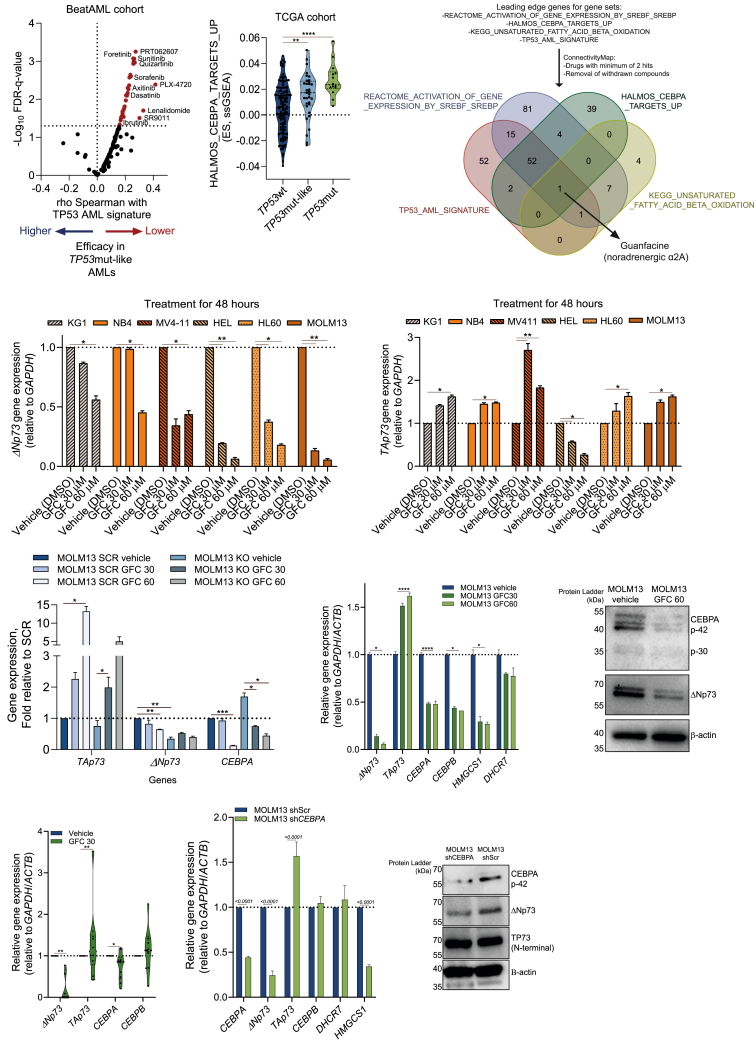
CEBPA controls ΔNp73 expression in AML cells (A) Spearman correlations between the TP53 AML signature and the *ex vivo* drug screening in the BeatAML cohort (122 drugs).^14^. Red and blue dots indicate resistance and sensitivity to drug-induced cell death in *TP53*mut-like AMLs, respectively. (B) Violin plots displaying the ES for the HALMOS_CEBPA_TARGETS_UP signature for AML patients included in the TCGA cohort (*n* = 173). Patients were categorized according to the *TP53* mutational status into *TP53*wt, *TP53*mut-like, and *TP53*mut. (C) Simplified schematic and Venn diagram analysis for drug repurposing discovery via cMAP analysis integrating the significant gene sets associated with *TP53*mut-like AMLs. (D and E) Relative mRNA expression levels of Δ*Np73* (D) and *TAp73* (E) at baseline and upon guanfacine (GFC) treatment (30 and 60 μM) in a panel of AML cell lines (48 h). (F) Relative mRNA expression levels of *TAp73*, *ΔNp73*, and *CEBPA* at baseline and upon guanfacine (GFC) treatment (30 and 60 μM) in MOLM13 SCR controls and KO cells (48 h) (*n* = 4). (G) Relative mRNA expression levels of *TP73* isoforms and *CEBPA/CEBPB* and its related targets (*HMGCS1* and *DHCR7*) at baseline and upon GFC treatment (30 and 60 μM) in MOLM13 cells (48 h) (*n* = 4). (H) Western blot analysis for CEBPA and ΔNp73 in total cell extracts from MOLM13 cells treated with GFC (60 μM, 48 h). (I) Relative mRNA expression levels of *TP73* isoforms and *CEBPA/CEBPB* and *ex vivo*-treated primary AML patients at baseline and upon GFC treatment (*TP53*mut/mut-like, 30 μM, 72 h) (*n* = 10). (J) Relative mRNA expression levels of the same targets as described in (G) in MOLM13 cells transduced with shRNA targeting the *CEBPA* gene and the scrambled control (*n* = 4). (K) Western blot analysis for CEBPA, ΔNp73, and total TP73 in total cell extracts from MOLM13 cells transduced with shScr (control) or the shRNA targeting the *CEBPA* gene (shCEBPA). Data are reported as mean ± SEM for (D)–(G), (I), and (J). The *p* values and cell lines are indicated in the graphs; ∗*p* < 0.05; ∗∗*p* < 0.01; ∗∗∗*p* < 0.001; ANOVA and Bonferroni post-test.

Till date, most drugs approved for AML treatment provide low efficacy in *TP53*-mutated AMLs, likely due to impaired activation of TP53 downstream signaling pathways (Figure 4A). ssGSEA of the TCGA/HOVON datasets and our cohorts indicated that patients with alterations in the TP53 signaling pathway (including deletion of the *TP53* locus on chromosome 17p, *TP53*mut, and *TP53*mut-like patients) exhibit gene expression programs enriched for processes such as “Halmos CEBPA targets up,” “LSC-up,” “TP53 AML signature,” “KEGG mitochondrial fatty acid oxidation of unsaturated fatty acids,” “REACTOME activation of gene expression/cholesterol biosynthesis by SREBF/SREBP,” and “Ferroptosis-up” (Figures 4B, S4A, and S4B). Interestingly, a subset of patients belonging to *TP53*wt/non mut-like subgroups (Figure S4A, highlighted in yellow) also displayed increased expression for CEBPA and SREBF/SREBP signaling, highlighting the molecular heterogeneity among AML patients. Similar results regarding the expression for the previously mentioned signatures were observed using a panel of *TP53*mut and KO MOLM13 cell lines generated by CRISPR-Cas9 editing in a previous study^29^ (Figure S4C), where a higher signature value was observed in the *TP53*-KO model.

One of the transcription factors predicted to bind to the enhancer region was CEBPA (Figure 3K), and CEBPA targets were also found to be upregulated in MOLM13-ΔNp73 OE cells and primary *TP53*mut/mut-like patient samples (Figures 2D and 4B). Using the Connectivity Map (cMAP) to identify Food and Drug Administration (FDA)-approved drugs targeting gene signature processes enriched in *TP53*mut/mut-like patients, we identified guanfacine (GFC) as a potential candidate to target this group of patients (Figure 4C). GFC has previously been identified as a compound with CEBPA inhibitory functions in AML cells.^30^ Treatment of AML cell lines with GFC significantly reduced the expression of *ΔNp73*, while restoring *TAp73* expression (Figures 4D and 4E). Furthermore, GFC treatment of MOLM13 enhancer-KO cells resulted in downregulation of *CEBPA*, which correlated with upregulation of *TAp73* but had no effect on *ΔNp73* levels (Figure 4F). This indicates that *CEBPA* may directly regulate *ΔNp73* expression through the intragenic region of *TP73*, whereas its influence on *TAp73* appears to be limited. Although GFC blocked cytokine-induced differentiation in MOLM13 cells, it had no significant effect on cell viability as a single agent across various AML cell lines (Figures S4D and S4E). Molecularly, GFC reduced the expression of *CEBPA*, *CEBPB*, and their target genes (*HMGCS1* and *DHCR7*) in MOLM13, HL60, and MV4-11 cells (Figures 4G, 4H, S4F, and S4G), which was validated in primary AML samples (Figure 4I). Genetic KD of *CEBPA* in MOLM13 cells phenocopied the effects of GFC leading to the downregulation of *ΔNp73* and *CEBPA* targets, while *TAp73* was upregulated (Figures 4J, 4K, and S4H). Together, these data suggest that *ΔNp73* expression is regulated by CEBPA that binds to the intragenic enhancer region, which can be targeted by GFC.

### Treatment with GFC overcomes drug resistance caused by ΔNp73-OE

**Figure 5:**
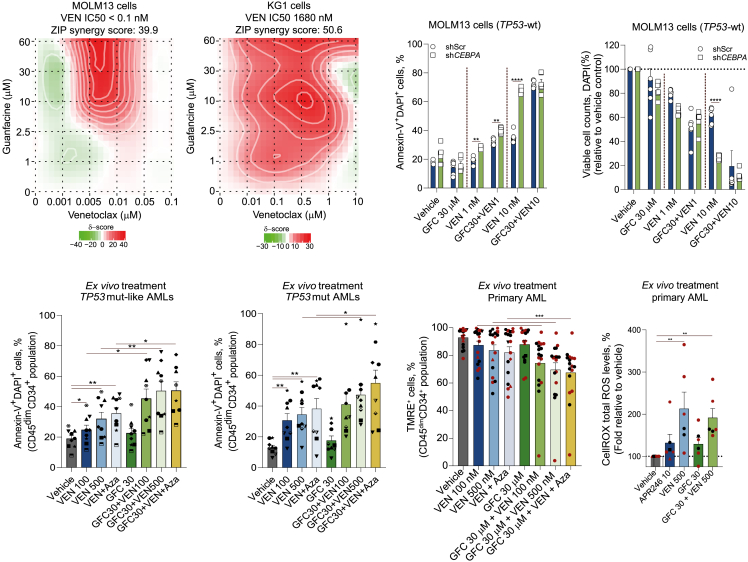
Pharmacological and genetic inhibition of CEBPA synergizes with VEN-induced apoptosis in TP53mut/mut-like AMLs (A) MOLM13 (VEN-sensitive) and KG1 (VEN-resistant) cells were treated for 72 h with increasing concentrations of VEN and GFC. Synergy was determined by Bliss coefficient (ZIP score >10 indicates synergism). (B and C) Drug-induced apoptosis (B) and viable cell counts (C) in MOLM13 shCEBPA/shScr cells treated with VEN alone or in combination with GFC (concentrations indicated in the plots, 72 h) detected by flow cytometry (*n* = 4). (D and E) Apoptosis was detected by flow cytometry in gated human CD45^dim^CD34^+^ (or CD117^+^ cells for CD34^−^ AMLs) of *ex vivo*-treated AML samples categorized as *TP53*mut-like (*n* = 8) (D) and *TP53*mut (*n* = 9) (E) in a co-culture system using an FITC-annexin V/DAPI staining method. Cells were treated with vehicle, VEN (100 and 500 nM), and VEN+Aza (VEN 100 nM + 5′ Aza 1.5 μM), in the presence or absence of GFC (30 μM) for 72 h. Bar graphs represent the mean ± SEM of all the independent patients screened; each point represents a patient. (F and G) Mitochondrial membrane potential (F) (measured by TMRE staining, *n* = 18) and total cytoplasmatic ROS levels (G) (measured using the CellROX Red probe, via flow cytometry, *n* = 6) for the data included in (D) and (E). *TP53*mut AMLs are depicted in red, and *TP53*mut-like AMLs are depicted in black. APR-246, eprenetapopt. Data are reported as mean ± SEM for (B)–(G). The *p* values and cell types are indicated in the graphs; ∗*p* < 0.05; ∗∗*p* < 0.01; ∗∗∗*p* < 0.001; ANOVA and Bonferroni post-test.

Since GFC treatment downregulated *ΔNp73* expression, we questioned whether combining GFC with standard-of-care therapies would enhance their efficacy in *TP53*mut/*TP53*mut-like AMLs. We treated a panel of 13 AML cell lines (including VEN-sensitive and VEN-resistant models) with the combination of GFC plus VEN. In both VEN-sensitive (MOLM13) and VEN-resistant (KG1) models, the combination of GFC plus VEN exhibited a strong synergic effect (Figure 5A). Treatment of CEBPA-KD AML models revealed increased drug sensitivity to VEN, with no additional effects observed for the GFC combinations (which were still observed in the control cells) (Figures 5B, 5C, S5A, and S5B). These results further support the notion that GFC drives increased cell death via CEBPA downregulation and de-repression of *TAp73* expression.

To test whether GFC combinations would enhance apoptosis induction in difficult-to-treat AML patients, we performed an *ex vivo* drug screen using either *TP53*mut-like (*ΔNp73*^high^) or *TP53*mut primary AML samples. While GFC treatment as a single agent exhibited limited cytotoxicity, the combination with VEN or VEN+Aza induced significant cell death (Figures 5D and 5E). Concordantly, we also observed a significant decrease in mitochondrial membrane potential upon GFC plus VEN or VEN+Aza treatment (Figure 5F), with increased total and lipid reactive oxygen species (ROS) generation (Figures 5G and S5C). Screening of GFC combination schemes in non-*TP53*-mutated/*TP53*mut-like patients, including those with *CEBPA*-mutant AML (typically associated with CEBPA loss of function), revealed no significant effect from the addition of GFC to cytotoxic therapy, suggesting that the effects of GFC are primarily mediated by CEBPA modulation in AML (Figure S5D). Finally, the combination of GFC with VEN showed no significant effects on normal CD34^+^ cells (Figure S5E), suggesting a favorable therapeutic window for this treatment therapy.

### Targeting ferroptosis represents a vulnerability in *TP53*mut-like patients

**Figure 6:**
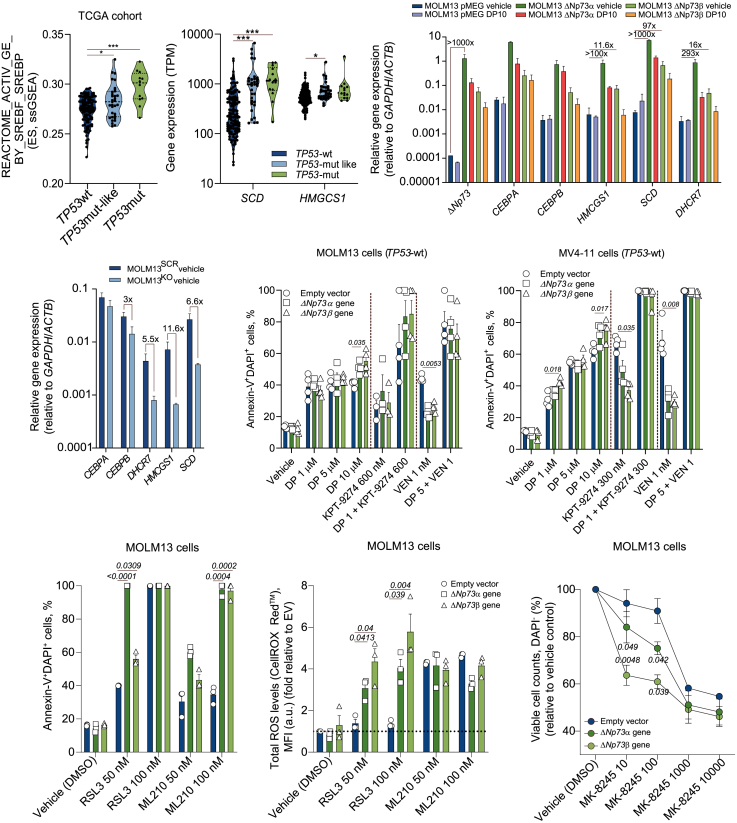
*TP53*mut/mut-like AMLs are associated with increased susceptibility to ferroptosis-induced cell death (A) Violin plots displaying the ES for the REACTOME_ACTIVATION_GENE_EXPRESSION_BY_SREBF_SREBP (left side) and the gene expression levels (transcripts per million, TPM) for the SREBP-related genes (*SCD* and *HMGCS1*) for AML patients included in the TCGA cohort (*n* = 173). Patients were categorized according to the *TP53* mutational status into *TP53*wt, *TP53*mut-like, and *TP53*mut. (B and C) Relative mRNA expression levels of Δ*Np73*, *CEBPA/CEBPB*, and its related targets at baseline and upon dipyridamole (DP) treatment (10 μM) in MOLM13 ΔNp73-OE/EV (pMEG) cells (48 h) (B) and at baseline in MOLM13-KO/Scr control cells (C) (*n* = 4). (D) Drug-induced apoptosis in MOLM13 and MV4-11 cells (ΔNp73-OE and EV control) treated with ferroptosis-related drugs KPT-9274 (NAMPT inhibitor^33^) and DP alone or in combination with VEN (concentrations indicated in the plots, 72 h) detected by flow cytometry. (E and F) Drug-induced apoptosis (E) and total ROS levels (F) in MOLM13 cells (ΔNp73-OE and EV control) treated with the GPX4 inhibitors RSL3 and ML210^31^ (concentrations indicated in the plots, 72 h) detected by flow cytometry. (G) Viable cell counts of MOLM13 cells (ΔNp73-OE and EV control) treated with the SCD inhibitor MK-8245 (concentrations indicated in the plots, 72 h) detected by flow cytometry (*n* = 4). Data are reported as mean ± SEM for (B)–(G). The *p* values and cell types are indicated in the graphs; ∗*p* < 0.05; ∗∗*p* < 0.01; ∗∗∗*p* < 0.001; ANOVA and Bonferroni post-test.

Recent studies suggested the involvement of CEBPA in the oxidative stress response and lipid metabolism, pathways closely implicated in ferroptosis.^31^^,^^32^ Activation of gene expression mediated by the SREBP/SREBF family was among the top pathways upregulated in *TP53*-mutant/mut-like AMLs (Figure 6A), suggesting an increase in lipid metabolism and cholesterol uptake in this group. Gene expression analysis of the ΔNp73-OE cell line confirmed increased expression of SREBP-related genes, which could be reduced with dipyridamole (DP) treatment, a compound previously reported as a negative modulator of the SREBP pathway^33^ (Figures 6B and S6A). Additionally, the SREBP-related genes *SREBF2*, *SPRING1*, *HMGCL*, and *HMGCR* were found to be bound by ΔNp73 in our MOLM13-ΔNp73 OE model, as shown in our ChIP-seq analysis (Figure 2G). Consistently, deletion of the ΔNp73 enhancer region was able to reduce the expression of SREBP-related genes (Figure 6C). Increased expression of SREBP target genes, including *SCD* and *HMGCS1*, was also seen in *TP53*mut and *TP53*mut-like AML patients (Figure 6A).

To determine whether *TP53*mut-like AMLs are more sensitive to ferroptosis-mediated cell death, we tested the effects of DP alone and in combination with VEN therapy in ΔNp73-OE models. As a positive control, we included the NAMPT inhibitor KPT-9274 previously reported to induce ferroptosis in AML.^33^ Both DP and KPT-9274 monotherapies induced significant cell death in ΔNp73-OE models. Additionally, the DP + VEN combination was strongly effective in overcoming ΔNp73-mediated drug resistance (Figures 6D and S6B), suggesting lipid/cholesterol metabolism as a potential vulnerability in *TP53*mut-like/mut patients.

To validate our observations, we treated ΔNp73-OE models with ferroptosis-inducing drugs RSL3 and ML210. Similar to GFC, treatment of ΔNp73-OE AML cells with RSL3 and ML210 significantly induced apoptosis (Figures 6E, S6C, and S6D). These effects were accompanied by increased levels of total ROS (Figures 6F, S6E, and S6F). Similar results were obtained with the SCD inhibitor MK-8245 (associated with ferroptosis induction^34^), causing a significant reduction in viable cell counts in ΔNp73-OE models even at lower dosages (Figures 6G, S6G, and S6H).

## Discussion

The therapeutic landscape of AML has significantly expanded in recent years beyond the traditional 3 + 7 regimen to include targeted therapies such as inhibitors of FLT3-ITD, IDH1/2, Menin, and BCL2 (VEN) and epigenetic therapies like HMAs.^35^^,^^36^ However, emerging resistance mechanisms continue to limit the efficacy of these treatments and remain a clinical challenge. Resistance in AML can arise through cell-intrinsic adaptation mechanisms or from pre-existing therapy-resistant leukemic stem cells (LSCs) with long-term self-renewal capacity.^37^^,^^38^^,^^39^ Cytogenetic and mutational factors, such as *TP53* mutation or chromosomal deletion (del17/17p), are also established contributors to resistance, conferring adverse prognoses due to increased stemness, an impaired DNA damage and apoptosis response, and dysregulated cell cycle control.^40^^,^^41^^,^^42^

While these cellular processes are often exacerbated in *TP53*mut patients, there are also patient subgroups with a dismal prognosis that are *TP53*wt and for whom explanations for their dismal prognosis have remained unknown. Here, we identify the existence of *TP53*wt AML patients who exhibit a molecular program comparable to *TP53*mut AML patients, as noted in two other recent studies.^43^^,^^44^ This subgroup, which we classified as *TP53*mut-like, is associated with drug resistance and poor outcomes, with enrichment for immature stem cell-like transcriptional programs, inflammation, cholesterol biosynthesis, and gene expression driven by SREBF/SREBP. These findings suggest a link between chronic inflammation and *TP53*mut leukemic progression. A specific feature of the *TP53*mut-like patient subgroup is the high expression of the truncated and transcriptionally inactive isoform *ΔNp73*, which inhibits recruitment of TP53 to the chromatin, thereby mimicking the molecular processes observed in *TP53*-mutated patients. Notably, and contrary to our expectations, overexpression of *ΔNp73* in *TP53*wt AML cells led to upregulation of *PMAIP1*, a classical TP53 transcriptional target. This observation suggests that *PMAIP1* can also be induced through TP53-independent mechanisms. In this context, transcription factors such as ATF4, c-MYC, FOXO3A, and DDIT3 (which is also upregulated in these cells) may mediate this response, potentially in response to elevated inflammatory signaling or cellular stress. Identifying ΔNp73 as a biomarker for this subgroup carries significant clinical implications, as it provides a single, actionable target compared to broader genetic signatures previously associated with *TP53*mut-like AML. While we and others^10^^,^^45^^,^^46^ identified that expression of the transcriptionally active full-length *TAp73* isoform in AML is comparable to healthy CD34^+^ cells, the expression of *ΔNp73* was significantly higher in AML patients, suggesting that the balance between *ΔNp73* and *TAp73* determines the oncogenic activity. Indeed, a high *ΔNp73*/*TAp73* ratio was linked to poor clinical outcomes in AML patients with favorable risk.^47^^,^^48^ Our data support the importance of incorporating *ΔNp73* assessment into diagnostic workflows, enabling more accurate risk stratification and guiding personalized treatment approaches.

Using chromatin accessibility data, we identified a functional role for a regulatory intragenic region of the *TP73* gene, which modulates *ΔNp73* expression and influences the response to standard-of-care (SOC) therapy in AML. Chromatin and epigenetic profiling identified this region as a critical enhancer-like element specifically linked to the upregulation of *ΔNp73* in AML cells. Ong et al.^26^ demonstrated enrichment of active histone modifications such as H3K27ac at this intragenic region in adult T cell leukemia/lymphoma, correlating with *TP73* expression and providing clonal advantages to these cells. Using functional experiments, we confirmed its essential role for *ΔNp73* transcriptional activation. *ΔNp73* expression driven by this regulatory element was associated with reduced apoptotic response to SOC therapies such as AraC and VEN, further underscoring its role in therapy resistance. These findings highlight the therapeutic potential of targeting this regulatory region to modulate *ΔNp73* expression and sensitize AML cells to existing regimens.

The transcription factor CEBPA was identified as a key regulator of *ΔNp73* expression in AML. CEBPA modulates the balance between monounsaturated fatty acids (MUFAs) and polyunsaturated fatty acids (PUFAs) in AML cells, driving lipid metabolic adaptations critical for therapy resistance.^30^^,^^31^^,^^49^ By favoring MUFA production, CEBPA reduces lipid peroxidation and oxidative damage, providing protection against ferroptosis, a form of regulated cell death driven by lipid peroxidation.^50^^,^^51^ This mechanism is particularly significant in the context of resistance to FLT3 inhibitors, as the altered lipid landscape enables leukemic cells to withstand oxidative stress induced by targeted therapies. Targeting CEBPA-driven metabolic rewiring could thus represent a strategy to overcome resistance in *FLT3*-mutant AML.^30^ We propose that high expression of *ΔNp73* creates a reliance on MUFA metabolism to facilitate lipid detoxification and protects leukemic cells against therapy-induced oxidative stress. In this scenario, ferroptosis emerges as a critical vulnerability in ΔNp73^high^ AML. We hypothesize that ΔNp73, through its regulation of MUFA-related genes, protects AML cells from ferroptosis, thereby supporting their survival under cytotoxic stress. This ferroptosis-susceptible phenotype is also observed in *TP53*mut AML, where ferroptosis induction has shown therapeutic potential in both preclinical and clinical studies, particularly with the *TP53*-reactivating compound APR-246.^52^^,^^53^ Notably, *CEBPA* levels are not upregulated in *TP53*mut/mut-like AML patients, suggesting that these subtypes finely regulate *CEBPA* levels to regulate lipid metabolism-related functions. Collectively, our findings support ferroptosis induction as a therapeutic approach not only for *TP53*mut but also for *TP53*wt/ΔNp73^high^ AML.

Building on these insights, we propose repurposing GFC, an FDA-approved drug for attention deficit hyperactivity disorder, as a ferroptosis-inducing agent for high-risk AML subgroups. GFC modulates *CEBPA* and *ΔNp73* expression, targeting the core metabolic dependencies of these cells. Its established safety profile and clinical availability offer a fast-track opportunity to improve outcomes in patients with *TP53*wt/ΔNp73^high^ AML and *TP53*mut AML. The synergy observed between GFC and VEN highlights its therapeutic potential in overcoming drug resistance in these difficult-to-treat patients.

While this study provides valuable insights, it has certain limitations. The precise mechanisms by which ΔNp73 modulates the MUFA:PUFA ratio and interacts with CEBPA-regulated gene networks remain to be elucidated. Additionally, while preclinical models demonstrated metabolic vulnerabilities and resistance mechanisms, these findings require validation in larger, clinically relevant patient cohorts. Lastly, potential off-target effects of ferroptosis-inducing agents like GFC and DP^54^^,^^55^ need further exploration to ensure therapeutic safety and efficacy. Although we demonstrated that ΔNp73 binds to several SREBP-related genes, the precise mechanism by which it regulates their expression remains unclear. Given that ΔNp73 lacks a TA domain, it is likely that additional cofactors or binding partners are required to mediate its regulatory effects.

Our study identifies a previously unidentified high-risk subgroup of *TP53*wt AML patients who exhibit *TP53*mut-like features, driven by the upregulation of *ΔNp73*. This upregulation blocks TP53 downstream signaling and promotes lipid/cholesterol metabolism. We further demonstrate that ΔNp73 and SREBF-related genes are regulated by the transcription factor CEBPA, which binds to a *TP73* intragenic enhancer region. Genetic KD or pharmacological inhibition of CEBPA using the FDA-approved drug GFC induces apoptosis in *TP53*mut-like AMLs. By establishing ΔNp73 as a critical driver of therapy resistance and poor prognosis in these patients and elucidating the role of CEBPA in regulating ΔNp73 expression, our findings reveal actionable vulnerabilities. Targeting this pathway with ferroptosis-inducing agents, such as GFC and DP, offers promising alternative treatment strategies for high-risk AML patients.

### Limitations of the study

Despite our multi-cohort and multi-model approach to functionally demonstrate the role of ΔNp73^high^ as a marker of a poor prognostic AML subtype that shares similar features with *TP53*mut patients, some limitations remain and require cautious interpretation. While in several contexts an upregulation of SREBP and its targets would prevent ferroptosis, it has been described that *TP53*mut AMLs are more sensitive to ferroptosis induction but also express higher levels of SREBP in line with our current observations. Our interpretation is that metabolic programs in *TP53*mut as well as *TP53*mut-like AMLs expressing ΔNp73 have changed such that these cells become more dependent on anti-ferroptosis machinery, which can be achieved via upregulation of SREBP and its targets, but this dependency also renders cells more sensitive to ferroptosis inducers. How metabolic programs change exactly as a consequence of *TP53* mutations or overexpression of ΔNp73 will require further investigation. Future targeted lipidomics and rescue experiments, for instance by modulating SCD or GPX4, are needed to further strengthen this point. Furthermore, we show correlations between ΔNp73, SREBP-related gene expression and sensitivity to ferroptosis induction, but we do not provide definitive causal proof that ΔNp73 directly drives MUFA/PUFA remodeling or that this remodeling solely underlies ferroptosis vulnerability. If ΔNp73 is indeed able to directly drive SREBP expression, it will be interesting to identify which mechanisms are involved as ΔNp73 itself lacks a TA domain. Additionally, *in vivo* combination treatment studies, for instance by evaluating the benefit of adding GFC to VEN or chemotherapy regimens, are warranted to confirm the translational relevance of our findings. Finally, even though GFC-induced phenotypes were phenocopied by KD of CEBPA, it is clear that multiple targets exist downstream of GFC, and future studies considering detailed pharmacodynamic profiles and safety studies are required before clinical translation.

## Resource availability

### Lead contact

Further information and requests for resources and reagents should be directed to and will be fulfilled by the lead contact, Jan Jacob Schuringa (j.j.schuringa@umcg.nl).

### Materials availability

All unique/stable reagents generated in this study are available from the lead contact with a completed materials transfer agreement (j.j.schuringa@umcg.nl).

### Data and code availability

• The ChIP-seq and RNA-seq experiments using modified MOLM13 cells (ΔNp73-OE and/or KO of the intronic region of the *TP73* gene) have been deposited at Gene Expression Omnibus repository (GEO) and is publicly available under the identifier GSE310074 as of the date of publication.
• All original code used to generate the data from this paper has been cited with the appropriate references.
• Any additional information required to reanalyze the data reported in this work paper is available from the lead contact upon request (j.j.schuringa@umcg.nl).

## Acknowledgments

This investigation was supported by Fundação de Amparo à Pesquisa do Estado de São Paulo (10.13039/501100001807FAPESP, grant #2013/08135-2, CNPq: 465539/2014-9). D.A.P.-M. received a fellowship from 10.13039/501100001807FAPESP (grant #2017/23117-1). I.W. received a fellowship from 10.13039/501100001807FAPESP (grant #2015/09228-0). L.Q., P.C., and D.R.S. were funded by 10.13039/100014013UKRI/10.13039/501100000265MRC grant #MR/R007608/1. A.R.L.-A. received a fellowship from Conselho Nacional de Desenvolvimento Científico e Tecnológico (10.13039/501100003593CNPq, grant #303914/2021-1 and grant #405918/2022-4). I.W. and D.A.P.-M. were sponsored by the Abel Tasman Talent Program (ATTP) of the Graduate School of Medical Sciences of the 10.13039/501100001721University of Groningen/10.13039/501100005075University Medical Center Groningen (UG/UMCG), the Netherlands.

## Author contributions

D.A.P.-M., C.O., I.W., E.M.R., and J.J.S. conceived and designed the study, performed the experiments, analyzed and interpreted the data, performed the statistical analyses, and drafted the article. A.T.J.W., V.v.d.B., F.M., D.S., N.K.v.d.M., S.M.H., T.M.B., P.C., and A.R.L.-A. performed the experiments, collected the data, and reviewed the paper. D.R.S. and L.Q. performed the experiments, performed statistical and bioinformatics analysis, and reviewed the paper. N.K.v.d.M., E.A., E.M.R., and G.H. provided patient samples and clinical data and reviewed the paper. D.A.P.-M., D.S., and J.J.S. conceptualized and generated the graphical abstract. All authors gave final approval of the submitted manuscript.

## Declaration of interests

The authors declare no competing interests.

## STAR★Methods

### Key resources table

**Table:** 

REAGENT or RESOURCE	SOURCE	IDENTIFIER
**Antibodies**		

Anti-Human CD45 FITC (1:50 dilution)	BioLegend	368508 RRID:AB_2566368
Anti-Human CD45 APC-Cy7 (1:100 dilution)	BioLegend	304014 RRID:AB_314402
Anti-Human CD14 PercP Cy5 (1:50 dilution)	BioLegend	301848 RRID:AB_2564059
Anti-Human CD117 APC (1:50 dilution)	BD Biosciences	550412 RRID:AB_398461
Anti-Human CD34 PE-Cy7 (1:50 dilution)	BioLegend	343516 RRID:AB_1877251
Anti-Human CD34 PE (1:50 dilution)	BD Biosciences	550761 RRID:AB_393871
Anti-Human CD38 APC (1:50 dilution)	BioLegend	303510 RRID:AB_314362
Anti-Human CD123 PE-Cy7 (1:50 dilution)	BioLegend	983702 RRID:AB_2749873
Anti-Human CD45RA BV421 (1:50 dilution)	BioLegend	304130 RRID:AB_10965547
Anti-human CD11b APC (1:50 dilution)	BioLegend	101212 RRID:AB_312795
Anti-human CD11b FITC (1:20 dilution)	Immunotools	21279113X2
Anti-human CD11b PE-Cy7 (1:100 dilution)	BioLegend	301322 RRID:AB_830644
Anti-mouse Ly-6A/E (Sca-1) APC (1:100 dilution)	BioLegend	108112 RRID:AB_313349
Annexin FITC (1:200)	Immunotools	31490013X2
Annexin APC (1:200)	Immunotools	31490016X2
Donkey anti-Rabbit (H + L) AF647	ThermoFisher	A32795 RRID:AB_2762835
Rabbit anti-Mouse (H + L) AF594	ThermoFisher	A27027 RRID:AB_2536090
Anti-GFP (rabbit polyclonal)	Abcam	ab290 RRID:AB_303395
Anti-TP53 (mouse monoclonal)	Santa Cruz Biotechnology	sc-126 RRID:AB_628082
Rabbit IgG control antibody, unconjugated	Sigma-Aldrich	I8140 RRID:AB_1163661
anti-TAp73 (mouse monoclonal)	Novus Biologicals	5B1288
anti-ΔNp73 (mouse monoclonal)	Santa Cruz Biotechnology	sc-70966 RRID:AB_1127552
Anti-CEBPA (mouse monoclonal)	Santa Cruz Biotechnology	sc-365318 RRID:AB_10846948
Anti-Beta Actin (mouse monoclonal)	Santa Cruz Biotechnology	Sc-47778 RRID:AB_626632

**Biological samples**		

Human AML bone marrow blast cells	University Medical Center Groningen	Ethical committee NL43844.042.13
Human cord-blood CD34^+^ cells	University Medical Center Groningen	Ethical committee NL43844.042.13
Human APL bone marrow blast cells	University of Sao Paulo	Ethical committee #13496/2005

**Chemicals, peptides, and recombinant proteins**		

4′,6-diamidino-2-phenylindole	Sigma-Aldrich	28718-90-3
Paraformaldehyde	Sigma-Aldrich	30525-89-4
RNAse	–	–
DNase I	Roche	11284932001
MgSO4	Sigma-Aldrich	M7506
Heparin	Sigma-Aldrich	60800-63-7
Verapamil hydrochloride	Sigma-Aldrich	152-11-4
Cytarabine	Sigma-Aldrich	147-94-4
Azacitidine; 5-AzaC; Ladakamycin	MedChemExpress	HY-10586R
Decitabine	MedChemExpress	HY-A0004R
Arsenic Trioxide	Sigma-Aldrich	1327-53-3
*All Trans* Retinoic Acid	Sigma-Aldrich	302-79-4
Midostaurin	Sigma-Aldrich	M1323
Quizartinib	Selleckchem	S1526
Gilteritinib (ASP2215)	MedChemExpress	HY-12432
Venetoclax	Selleckchem	S8048
KPT-9274 (ATG-019)	Selleckchem	S8444
Guanfacine hydrochloride	MedChemExpress	HY-17416
Dipyridamole	MedChemExpress	HY-B0312R
MK-8245	MedChemExpress	HY-13070
RSL3 ((1S,3R)-RSL3)	MedChemExpress	HY-100218A
ML-210	MedChemExpress	HY-100003
Eprenetapopt (APR-246)	MedChemExpress	HY-19980
Human Interleukin 6	Peprotech	200–06
Human Interleukin 3	Peprotech	200–03
Human Granulocyte colony-stimulating factor	Peprotech	300–23
Human Thrombopoietin	Amgen	–
Human Granulocyte/Macrophage colony stimulating factor	Amgen	–
β-mercaptoethanol	Merck Sharp & Dohme BV	60-24-2
SsoAdvanced Universal SYBR® Green Supermix	BioRad	1725274
iScript cDNA synthesis Kit	BioRad	1708891BUN
Tetramethylrhodamine, Ethyl Ester, Perchlorate	Thermofisher	T669
CellROX™ Deep Red Reagent	Thermofisher	C10422
BODIPY™ 581/591 C11 undecanoic acid (Lipid Peroxidation Sensor)	Thermofisher	D3861
FcR blocking reagent	Mylteni Biotech	130-059-901
Protein G Dynabeads	Invitrogen	10004D

**Critical commercial assays**		

CD34 MicroBead Kit, human	Miltenyi Biotech	130-046-703
NucleoSpin tissue kit	Machery-Nagel	740952
RNeasy micro kit	Qiagen	74004
QIAquick PCR purification kit	Qiagen	28106
Amicon Ultra-15 Centrifugal Filter Unit – 100 KDa	Merck	UFC910024
CD117 MicroBeads Kit, Human	Miltenyi Biotec	130-091-332
CD3 MicroBeads, Human	Miltenyi Biotec	130-050-101
MethoCult™	Stemcell	H4435
FuGENE HD Transfection Reagent	Promega	E2312
KAPA RNA HyperPrep Kit with RiboErase (HMR)	Roche	08098131702

**Deposited data**		

Raw and analyzed RNA-seq data from MOLM13 ΔNp73-OE and TP73 intronic region KO	This paper – Table S3	GEO: GSE310074
Raw and analyzed ChIP-seq data RNA-seq from MOLM13 ΔNp73-OE	This paper – Table S4	GEO: GSE310074
Transcriptomic analysis of the TCGA AML cohort	Ley et al.^13^	https://www.cbioportal.org/
Transcriptomic analysis of the BeatAML cohort	Tyner et al.^14^	http://www.vizome.org/
Transcriptomic analysis of the HOVON AML cohort	de Jonge et al.^16^; Verhaak et al.^17^	GEO: GSE6891
DNaseI-hypersensitive profiles of genetic distinct AML subclones	de Boer et al.^20^	GEO: GSE117667; https://proteomecentral.proteomexchange.org/cgi/GetDataset?ID=PXD030463
Transcriptomic analysis of MOLM13 cells with AML-related *TP53* mutations	Boettcher et al.^29^	GEO: GSE131592
Cancer cell line encyclopedia datasets (CCLE)	Broad institute	https://depmap.org/portal/ccle/
Label Free proteome on primary AML blasts (CD34^+^)	de Boer et al.^20^	PXD030463
DNaseI-hypersensitive profiles of AML samples	Blueprint epigenome	https://www.blueprint-epigenome.eu/

**Experimental models: Cell lines**		

MOLM13 (male origin)	DSMZ	ACC 554 RRID:CVCL_2119
MV4-11 (male origin)	ATCC	CRL-9591™ RRID:CVCL_0064
HL60 (female origin)	ATCC	CCL-240™ RRID:CVCL_0002
OCI-AML3 (male origin)	DSMZ	ACC 582 RRID:CVCL_1844
NB4 (female origin)	Harvard Medical School	Prof. Pier Paolo Pandolfi RRID:CVCL_0005
NB4-R2 (female origin)	Harvard Medical School	Prof. Pier Paolo Pandolfi
NB4-ATO resistant (female origin)	University of Rome Tor Vergata	Prof. Maria T Voso
MS-5 (male origin)	DSMZ	ACC 441 RRID:CVCL_2128
Lenti-X 293T™	Takara	CRL-3216
AS-E2 (male origin)	Nagasaki University School of Medicine	Dr. M. Tomonaga
KBM7 (male origin)	Brummelkamp lab	Dr. Thijn Brummelkamp RRID:CVCL_A426
Kasumi-1 (male origin)	DSMZ	ACC 220 RRID:CVCL_0589
HEL (male origin)	DSMZ	ACC 11 RRID:CVCL_0001
KG1 (male origin)	DSMZ	ACC 14 RRID:CVCL_0374
TF1 (male origin)	DSMZ	ACC 334 RRID:CVCL_0559
U937 (male origin)	DSMZ	ACC 5
THP1 (male origin)	ATCC	TIB-202™
K562 (female origin)	ATCC	CCL-243
OCI-AML2 (male origin)	DSMZ	ACC 99 RRID:CVCL_1619

**Experimental models: Organisms/strains**		

NOD.Cg-Prkdcscid Il2rgtm1Wjl Tg(CMV-IL3,CSF2,KITLG)1Eav/MloySzJ (NSGS mice)	The Jackon Laboratory	RRID: IMSR_JAX:013062
C;129S4-Rag2tm1.1Flv Csf1tm1(CSF1)Flv Csf2/Il3tm1.1(CSF2,IL3)Flv Thpotm1.1(TPO)Flv Il2rgtm1.1Flv Tg(SIRPA)1Flv/J (MISTRG mice)	University of Zurich and University Hospital Zurich	Prof. Markus G Manz RRID: IMSR_JAX:017712

**Oligonucleotides**		

gRNA primers for intragenic TP73 region knockout	Table S6	–
cDNA primers for gene expression analysis	Table S6	–
ChIP-qPCR primers	Table S6	–

**Recombinant DNA**		

pCMV-TurboGFP_shCEBPA SMARTvector Lentiviral shRNA (plasmid)	Dharmacon reagents	V3SH11240-224846075
pMSCV-EGFP-Puro-ΔNp73α/ΔNp73β (plasmid)	Lucena-Araujo et al.^56^	GFP fusion for ΔNp73 isoforms

**Software and algorithms**		

FlowJo v10.0.6	Treestar	http://www.flowjo.com/RRID:SCR_008520
Prism 9	GraphPad	http://www.graphpad.com/
SPSS Statistical package 19.1	IBM	https://www.ibm.com/
RStudio	CRAN	www.r-project.org
GSEA 4.0.1	Broad Institute	https://software.broadinstitute.org/gsea/RRID:SCR_003199
Single sample gene set enrichment analysis (ssGSEA)	Barbie et al.^57^	https://www.genepattern.org/modules/docs/ssGSEAProjection/4#gsc.tab=0
WashU Epigenome Browser	Li et al.^58^	https://epigenomegateway.wustl.edu/browser/RRID:SCR_006208
Elysium	Lachmann et al.^59^	https://maayanlab.cloud/cloudalignment/elysium.html
Morpheus	Broad Institute	https://software.broadinstitute.org/morpheus RRID:SCR_014975
Cytoscape 3.10.2	–	http://apps.cytoscape.org/apps/bingo RRID:SCR_003032
Synergy finder	Ianeviski et al.^60^	https://synergyfinder.fimm.fi/
Bowtie v2.3.1	Langmead and Salzberg et al.^61^	http://bowtie-bio.sourceforge.net/bowtie2/index.shtml RRID:SCR_016368
MACS v1.4.2	Zhang et al.^62^	http://liulab.dfci.harvard.edu/MACS/RRID:SCR_013291
Adobe Illustrator	Adobe	https://www.adobe.com/nl/RRID:SCR_010279
JBrowse2	JBrowse	https://jbrowse.org/jb2/RRID:SCR_001004
Connectivity Map – Clue	Broad Institute	https://clue.io/RRID:SCR_015674

### Experimental model and study participant details

#### Study approval and human patient samples

Peripheral blood (PB) and bone marrow (BM) samples of AML patients (*n* = 46, average age = 56.7 years, range 18–69.9 years; 53% female) were studied (for proteomic/transcriptomic studies) and *ex vivo* evaluation after informed consent and protocol approval by the Medical Ethical committee of the UMCG in accordance with the Declaration of Helsinki (protocol #NL43844.042.13). Neonatal cord blood (CB) was obtained from healthy full-term pregnancies from the Obstetrics departments of the University Medical Center and Martini Hospital in Groningen, The Netherlands, after informed consent. Peripheral blood mononuclear cell derived CD34^+^ stem cells (PBMSCs) and CB derived CD34^+^ cells were isolated by density gradient separation (Ficoll) (Sigma-Aldrich), followed by a hematopoietic progenitor magnetic associated cell sorting kit from Miltenyi Biotech (#130-046-702) according to the manufacturer’s instructions. All CD34^+^ healthy cells were pre-stimulated for 24-48h prior to experimental use. CB derived cells were pre-stimulated with StemlineII medium (SigmaAldrich; #S0192), 1% penicillin/streptomycin (PS) supplemented with SCF (255-SC, Novus Biologicals), FLT3 ligand (FLT3-L, Amgen) and N-plate (TPO) (Amgen) (all 100 ng/mL). PBMSC CD34^+^ cells were pre-stimulated with StemlineII, 1% PS, 20% fetal bovine serum (FBS) along with SCF, FLT3-L, N-plate (all 100 ng/mL) and IL-3 (Sandoz) and IL-6 (both 20 ng/mL). Primary AMLs were grown on MS5 stromal cells with G-CSF (Amgen), N-Plate and IL-3, all 20 ng/mL.

#### Study approval for *in vivo* experiments

BM samples of APL patients used in for *in vivo* experiments were studied after informed consent and protocol approval by the Ethical Committee in accordance with the Declaration of Helsinki (registry #12920; process number #13496/2005; CAAE: 155.0.004.000–05 and CAAE: 819878.5.1001.5440). Mononuclear cells (MNCs) were isolated via Ficoll separation and cryopreserved. For the *in vivo* experiments using the NSGS model (APL samples), all animals were housed under specific pathogen free conditions in individually ventilated cages during the whole experiment. The animals were maintained according to the Guide for Care and Use of Laboratory Animals of the National Research Council, USA, and to the National Council of Animal Experiment Control recommendations. All experiments were approved by the Animal Ethics Committee of the University of São Paulo (protocols #176/2015 and #095/2018). Eight weeks old female NSGS (NOD.Cg-Prkdcscid Il2rgtm1Wjl Tg(CMV-IL3,CSF2,KITLG)1Eav/MloySzJ – for primary APL samples) or MISTRG (C;129S4-Rag2tm1.1Flv Csf1tm1(CSF1)Flv Csf2/Il3tm1.1(CSF2,IL3)Flv Thpotm1.1(TPO)Flv Il2rgtm1.1Flv Tg(SIRPA)1Flv/J) mice were used for the transplant experiments. NSGS mice were purchased from the Jackson Laboratory and the MISTRG mice were kindly provided by prof. Alex Theocharides and prof. Markus Manz (University of Zurich and University Hospital Zurich, Zurich, Switzerland). Mice used in the experiment had an average weight of 24.2 g (±2.92 g). Mouse experiments were performed in accordance with national and institutional guidelines. For the *in vivo* experiments using primary AML samples, MISTRG mice were also housed under specific pathogen free conditions as used for the NSGS mice. All experiments were approved by the Animal Ethics Committee of the University Medical Center Groningen (protocol #2316947-01-001).

#### Cell lines

All cell cultures were maintained in a humidified atmosphere at 37°C with 5% CO_2_. Mycoplasma contamination was routinely tested. Leukemia cell lines were authenticated by short tandem repeat analysis. Cells were obtained from their correspondent biobank sources and were cultured according to the guidelines offered by the supplier.

### Method details

#### Transcriptomic and metabolomic analysis in AML cell lines and AML cohorts

The RNA sequencing and metabolomic analysis were performed on 13 AML cell lines at the Broad Institute included into the Cancer Cell Line Encyclopedia dataset.^23^^,^^63^^,^^64^ Transcriptomic data from the TCGA,^13^ BeatAML^14^ and HOVON (GSE6891) AML cohorts were retrieved via the cBioPortal platform^65^ (for TCGA and BeatAML) and the Gene expression omnibus (GEO) portal (for the HOVON cohort).

#### Development of a TP53 AML signature

Differential gene expression analysis was performed comparing patients with *TP53*wt versus *TP53*mut AML, using the TCGA^13^ and BeatAML^14^ cohorts. Up-regulated genes in *TP53*mut AML patients from both datasets were cross-compared, and the top 20% of genes with higher expression in *TP53*mut AMLs (157 genes, Table S2) were used to build the TP53 AML signature. Using single sample gene set enrichment analysis (ssGSEA, an extension from GSEA analysis), we generated enrichment scores (ES) for the TP53 AML signature and all the other 35k signatures present in the MSigDB^66^ portal patients from both TCGA and BeatAML cohorts, and included the HOVON cohort (GSE6891)^16^^,^^17^ (Table S3). Using unsupervised clustering analysis,^67^ we grouped the AML patients based on the ES values for the TP53 AML signature in addition to 65 signatures related to normal and malignant hematopoiesis and TP53 signaling pathway (Table S1). This analysis identified a group of *TP53*wt patients with a similar transcriptional program to *TP53*mut patients, which were called *TP53*mut-like AMLs. Internal validation was performed using a non-parametric bootstrap procedure with 1,000 resamplings to get estimates of ES values for the TP53 AML signature between the different groups corrected for overfitting.

#### *Ex vivo* drug screening in primary AML samples

Cryopreserved MNC fractions of AML patients were thawed and prepared as previously described,^68^ and resuspended in IMDM +20% FBS, +20 ng/mL of G-CSF, IL-3 and N-plate. Cells were plated at a cellular density of 1.5 million cells/mL for 48 h, to remove cellular debris that remained after the thawing procedure. For the *ex vivo* drug screening, cells were washed once in IMDM +20% FBS and plated at 1.5 x 10^5^ cells/mL in 48-well plates and treated with a dose-range of the different compounds (described in the Fig. legends) used to evaluate the cytotoxic effects on leukemic blasts. To analyze the cytotoxicity in the different fractions of the bulk treated AML cells, treated MNCs were blocked with human FcR blocking reagent (Miltenyi Biotec) for 5 min and stained with the following antibodies: CD45-APC-Cy7, TMRE, CD14-PerCP, CD34-PE-Cy7 (or CD117-PE-Cy7 for CD34^−^ samples), and CD11b-APC for 20 min at 4°C. After incubation, cells were washed once in PBS+2% FBS and at the end resuspended in IMDM +20% FBS supplemented with 10% of Ca^2+^ buffer (10X, BD biosciences, CA, USA) plus Annexin-V FITC (Biolegend, CA, USA) and the viability marker DAPI. For total reactive oxygen species (ROS) and for lipid ROS formation measurements, the CellROX DeepRed (removing CD11b-APC and replacing CD45-APC-Cy7 for CD45 FITC and CD34-PE-Cy7 for CD34 PE) and BODIPY C11 probes (removing the Annexin V FITC and the TMRE probe) (ThermoFisher) were used, respectively. Fluorescence was measured on the BD LSRII and analyzed using Flow Jo (Tree Star, Inc). The apoptosis induction, modulation of the mitochondrial membrane potential and the levels of total cytoplasmatic and lipid ROS were evaluated in the leukemic blast population (CD34^+^ or CD117^+^). For synergy analysis, ZIP scores were calculated using the SynergyFinder 3.0 tool.^60^ Combination treatment was considered synergistic when ZIP >10 and antagonistic when ZIP < - 10^30^.

#### Flow cytometry

Cryopreserved MNC fractions of AML patients were thawed, resuspended in newborn calf serum (NCS) supplemented with DNase I (20 Units/mL), 4 μM MgSO_4_ and heparin (5 Units/mL) and incubated at 37°C for 15 min (min). To analyze the hematopoietic stem progenitor cell (HSPC) populations of the AML bulk samples, 5x10^5^ mononuclear cells were blocked with human FcR blocking reagent (Miltenyi Biotec) for 5 min and stained with the following antibodies: CD45-FITC, CD34-PE, CD38-BV421, CD11b-PECy7, CD14 PerCP, Sca-1-APC (to exclude MS5 cells) and viability marker 7-AAD for 20 min at 4°C. Fluorescence was measured on the BD LSRII or FACS Symphony A5 and analyzed using Flow Jo (Tree Star, Inc). For each sample a minimum of 20000 events were acquired inside the SSC-A^low^CD45^dim^7-AAD^-^Sca-1^-^ population.

#### *In vivo* APL and AML xenotransplant

For the APL models ten different APL mononuclear cells (clinical characteristics previously published elsewhere^68^) were depleted for CD3^+^ cells and transduced twice with empty vector (EV, pMEG) or ΔNp73-OE vector (multiplicity of infection, MOI >50) using Retronectin-coated plates (Takara). Forty-eight hours post transduction, GFP levels were checked by flow cytometry (EV (mean ± Standard Deviation): 42.1 ± 6.3% and ΔNp73: 5.8 ± 0.9%) and 1 x 10^6^ transduced cells were directly injected into the tibia of the animals (*n* = 10 for each group). For the AML models, mononuclear cells from three independent AML patients were processed as described for the APL models. A total of 1.5x10^5^ sorted cells (GFP^+^) were transplanted via tail vein in sub-lethally irradiated MISTRG mice (1 Gy), 24h post-irradiation. Human CD45^+^ levels were measured regularly in blood obtained by sub-mandibular bleeding and mice were sacrificed after engraftment confirmation (12 weeks). Cells from the mouse organs including BM and spleen were isolated and analyzed for presence of GFP expression (transduced cells). Inside the population GFP^+^, we evaluated the presence of human APL blast, defined by the expression markers: CD45^+^CD117^+^CD33^+^HLADR^−^CD19^−^ and human myeloid committed cells, defined by CD45^+^CD117^−^CD33^+^, by flow cytometry. For the AML models, human engraftment was determined by positivity for GFP and CD45. All antibodies used for the staining were incubated following the manufacturer’s instructions. In parallel, cytospin preparations stained with May-Grünwald-Giemsa (MGG) were used to evaluate morphological changes. Left over cells from BM were sorted for GFP^+^CD45^+^CD117^+^CD33^+^ cells, to perform the *ex vivo* cultures and cryopreserved and stored in liquid nitrogen.

#### Western blot analysis

Equal amounts of protein were used as total extracts, followed by SDS-PAGE and Western blot analysis with the indicated antibodies. For imaging the SuperSignal West Dura Extended Duration Substrate System (Thermo Fisher Scientific, USA) and Gel Doc XR^+^ system (Bio-Rad, Hercules, CA, USA) were used. Antibodies against ΔNp73 (sc-70966), anti-TP73 (5B1288), TP53 (sc-126), CEBPA (sc-365318) and β-actin (sc-47778) were obtained from Santa Cruz Biotechnology (San Jose, CA). All membranes were incubated with a primary antibody following manufacturer’s instructions.

#### MOLM13 RNA sequencing and analysis

RNA samples for sequencing were prepared for transduced MOLM13 ΔNp73-OE, MOLM13-KO and MOLM13 SCR control cells plated at the same cell density (1x10^5^ cells/mL – 24 well plate) for 48 h. Cells were collected and viable cells were isolated for posterior RNA extraction. Total RNA was isolated using the RNeasy Mini Kit from Qiagen (Venlo, The Netherlands) according to the manufacturer’s recommendations. The obtained cDNA fragment libraries were sequenced on an Illumina NextSeq500 using default parameters (25M reads per sample). Sequencing reads were mapped to Hg38 with STAR version 2.7.3a^69^ using the default parameters filtered for uniquely mapping reads with the following modifications: ‘--outFilterType BySJout --outFilterMultimapNmax 20 --outFilterMismatchNoverLmax 0.04 --outSAMtype BAM sorted --outSJfilterReads Unique --chimSegmentMin 20’. Read counts were normalized as counts per million (CPM) and log2 transformed (Log2CPM). We used a filtering approach to eliminate non-expressed or marginally expressed genes in ENSEMBL annotation. We retained genes that had a CPM >1 in at least half of the samples of at least one of the experimental conditions considered. Thus, we retained 17,928 genes in our analysis (Table S4). We generated gene expression profiles by computing differential expressed genes (DEG), computing the log2-fold changes (Log2FC), *p*-values of differential expression (Wilcoxon), and the false discovery rate (FDR)–adjusted *p*-values (Benjamini and Hochberg) of DEG in all the profiles.^70^ The statistical significance was set as FDR <0.05. Differentially expressed genes were clustered using unsupervised hierarchical clustering with Euclidean distances (complete).^67^

#### ChIP-seq procedure and data analysis

##### ChIP experiment

Chromatin immunoprecipitation was performed as described previously.^71^^,^^72^ Five million MOLM13 cells transduced with EV or ΔNp73-OE were equally plated and incubated for 24 h at 37°C, 5% CO_2_. Cells were counted and equal cell numbers from each cell type were crosslinked. The following antibodies were used: anti-p53 (Santa Cruz biotechnologies, SCT, sc-126), anti-TAp73 (Novus Biologicals, 5B1288), anti-ΔNp73 (SCT, sc-70966), and IgG (i8141, Merck). Sequencing libraries were generated using the KAPA Hyper Prep Kit (Roche Sequencing and Life Sciences) according to manufacturer’s protocol and sequenced on an Illumina NextSeq500 using default parameters.

##### Alignment

ChIP-seq data analysis was done as previously described.^72^ In short, combined reference genomes were generated for human (hg38). Obtained paired-end reads were aligned to the metagenome using Burrows-Wheeler Aligner (BWA) with default settings. Aligned reads were further processed using SAMtools.

##### Visualization of tracks

To visualize the tracks bigwig files were generated by determining the total number of overlapping fragments at each position in the genome using BEDtools genomecov. The coverage was scaled using the calculated normalization factors. Subsequently, BedGraph files were converted to BigWig files using UCSC bedGraphToBigWig. Tracks were visualized using the Jbrowse2 software (https://github.com/GMOD/jbrowse-components).

##### Peak calling and further processing

Peaks were called using MACS2 with estimated fragment size and broad settings. To be able to compare coverage from different samples peaks were concatenated and merged per antibody. For every track read counts were generated and the coverage was normalized using the normalization factor calculated before. Heatmaps and average plots were generated using ngs.plot. Average plots were generated +/− 5kb of the TSS. Data displayed in Table S5.

##### Gene ontology (GO) and gene set enrichment analyzes (GSEA)

Gene ontology (GO) was evaluated using the gene ontology resource (http://geneontology.org/) and the BinGO plugin using the Cytoscape software v3.8.2 (NIGMS, USA). For the proteomic datasets, protein expression was correlated with the ΔNp73 mRNA levels, and ranked lists based on the Pearson correlation values were used to perform the GSEA analysis. All genes from the RNA-seq of the different experimental groups (ΔNp73^high^ versus ΔNp73^low^; MOLM13 ΔNp73-OE versus EV control) cohort were pre-ranked according to their differential expression (fold change). Enrichment scores (ES) were obtained with the Kolmogorov-Smirnov statistic, tested for significance using 1000 permutations, and normalized (NES) to consider the size of each gene set. As suggested by the GSEA, a false discovery rate (FDR) cut-off of 25% (FDR q-value <0.25) was used.^66^ Data visualization was performed with the ClustVis platform.^67^ ssGSEA enrichment scores were generated using R packages circlize and matrixStats. In summary, the selected list of gene sets from the MSigDB platform was used as input and ES per terms and per condition were acquired based on expression data for genes comprised in the specific term.

##### Lentiviral vectors and lentivirus production

Recombinant lentivirus to perform the overexpression of ΔNp73 isoforms alpha (α) and beta (β) in the various AML models was previously generated using the pMEG backbone.^56^ To perform the genetic knockdown of the *CEBPA* gene, a sequence for shCEBPA (shCEPA – V3SH11240-224846075; Dharmacon Reagents) was generated using different lentiviral backbone plasmids, in Lenti-X 293T cells according to the three-plasmid packaging procedure as previously described.^70^^,^^73^ Lentiviral particles were concentrated using Amicon Ultra-15 centrifugal filter unit columns (Merck, CA, USA). Cells were sorted based on their GFP protein expression and posteriorly used for *in vitro* assays. The efficiency of infection was further confirmed by flow cytometry. A shRNA sequence that does not target human genes (referred to as scrambled) was used as a control.

#### Generation of CRISPR/Cas9 deletion of TP73 region

##### 3xNLS-SpCas9 expression and purification

3xNLS-SpCas9 was purified essentially according to the method previously described, with some modifications.^74^ The pET-21a_3xNLS-SpCas9 vector (#114365) was obtained from Addgene, transformed into Rosetta 2(DE3)pLysS competent cells, and cultured under antibiotic selection in LB and TB media with IPTG induction. The culture was harvested, flash-frozen, and stored before purification. For protein isolation, the lysate was treated with lysozyme, PMSF, and DNase, followed by centrifugation and affinity purification using Ni-NTA agarose. The eluate was further purified via cation exchange chromatography, desalted, and concentrated to 5 mg/mL. Aliquots of the purified protein were flash-frozen in liquid nitrogen and stored at −80°C for future use.

##### Guide RNA selection

The online platform Benchling (www.benchling.com) was used to design guide RNA sequences for the intragenic region of the *TP73* gene, located +24Kb from the TSS. Two different gRNAs were selected based on high on-target and off-target scores. gRNA sequences are listed in Table S6.

##### sgRNA preparation

sgRNA was made by *in vitro* transcription of a dsDNA PCR product. In short: a DNA template was made by oligo assembly using a set of three generic oligos (Sp6-forward, scaffold oligo and Sp6-reverse) and one guide specific oligo. PhusionII HF polymerase (Thermo Scientific, Bleiswijk, the Netherlands) was used to amplify the DNA template.

##### Procedure

RNP complexes were formed *in vitro* by incubating 2.4 μg of sgRNA with 4 μg of SpCas9 for 15 min at room temperature. MOLM13 and HL60 cells (0.5 x 10^6^) were washed once with PBS and resuspended in 20 μL of “K562 electroporation buffer” (88 mM KH_2_PO_4_, 14 mM NaHCO_3_, 12 mM MgCl_2_, 2 mM glucose, and 6 mM ATP, pH 7.4). The RNP complex, along with 2 μg of ssODN, was added to the cell suspension and transferred to a 16-well electroporation cuvette strip. Electroporation was performed using an Amaxa 4D device (Lonza, Geleen, the Netherlands) with program CA137, and the cells were immediately transferred to 4 mL of fresh RPMI-1640 medium supplemented with 20% FBS.

Genomic DNA was isolated from the bulk-transfected cells, and the percentage of successfully mutated DNA was estimated using qPCR with primers specific to the mutated bases. Approximately 48 single-cell clones were expanded in 24-well plates, and genomic DNA was extracted from a portion of each clone. qPCR analysis identified several potential knockout (KO) clones. Four clones with homozygous deletion of the enhancer region, as confirmed by the template, were selected, expanded, and pooled for subsequent experiments.

##### Gene expression analysis by qPCR

Real-Time quantitative PCR assays were performed in triplicate using sample-derived RNA which was reverse transcribed using the iScript cDNA synthesis kit (Bio-Rad) on CFX384 Touch Real-Time PCR Detection System (Bio-Rad). The reaction solution was prepared by combining the SsoAdvanced SYBR Green Supermix (Bio-Rad) and 320 nM each of primers. Negative controls without template were run for each gene. At the end of the amplification process, the amplification specificity of the gene was assessed by a melting curve between 55°C and 95°C. The efficiency of all used primers was higher than 97%. Importantly, the same reference cDNA (from NB4 and THP1 cells) was used as an internal control in all experiments to ensure that the results of different experiments could be comparable. Following standardization between different runs, the relative gene expression for the target genes was obtained using the comparative cycle threshold (ΔCt) method, and the results were expressed using 2^−ΔΔCt^, in which ΔΔCt = ΔCt ^target cell^ – ΔCt ^internal control^. The *ACTB*, *GAPDH* and *RPL30* were used as housekeeping genes. Primer sequences were published elsewhere^68^^,^^70^ and provided here (Table S6).

##### *In vitro* primary AML cell proliferation

Cryopreserved MNC fractions of AML patients were thawed as described in the section “Flow cytometry”. CD34^+^ cells were isolated from primary AML patients on the autoMACS using a magnetically activated cell-sorting progenitor kit (Miltenyi Biotech). In case of *NPM1* mutated AMLs with CD34 expression <1%, the CD117^+^ blast cells were isolated.

A total of 1x10^5^-2.5x10^5^ primary AML were lentivirally transduced with *ΔNp73* overexpression constructs and the empty vector control (pMEG, EV), cultured on MS-5 cells, for 35 days. MS-5 cells were plated on gelatin-coated culture flasks and expanded to form a confluent layer (above 70% of confluence). The co-cultures were performed in Gartner’s medium consisting of IMDM (Thermo Scientific) supplemented with 20% FBS, 1% penicillin and streptomycin, 2 mM glutamine (Gibco), 57.2 mM β-mercaptoethanol (Merck Sharp & Dohme BV), and 20 ng/mL G-CSF, N-plate (TPO), and IL-3. Co-cultures were grown at 37°C and 5% CO_2_ and demi-populated after counting if necessary. Cell proliferation was assessed with a hemocytometer until 35 days of co-culture and cross validated by counting the viable cell population (DAPI^−^ cells), which were CD45^dim^ by flow cytometry evaluation using the NovoCyte Quanteon System (Agilent, CA, USA).

#### Generation transduced healthy CD34^+^ cells

PBMCs were isolated by a density gradient from CB. MNCs were washed once at 450g with PBS-EDTA (5 mM) and resuspended in 300 μL of PBS. Next, 100 μL of FcR blocking reagent and 100 μL of CD34 MicroBeads (Miltenyi Biotech) were added to the suspension and incubated for 30 min at 4°C. After incubation cells were washed for 10 min at 450g and resuspended in 2 mL of PBS–EDTA (5 mM). Cells were passed through a cell strainer (70 μm) and isolated by magnetic separation on the autoMACS (Program – Possedels, Miltenyi Biotech). The purity of the isolated cells was routinely evaluated by FACS and in the range of 85%–95%.

CB isolated CD34^+^ were next expanded in Stemcell II medium supplemented with 100 ng/mL SCF, 50 ng/mL FLT3-Ligand, 30 ng/mL GM-CSF and 10 ng/mL IL-6. Two days later, cells were collected and transduced with concentrated virus containing the pMEG (EV) and the pMEG-ΔNp73 overexpression constructs. After transduction the cells were cultured in IMDM supplemented with 20% FBS and 10 ng/mL SCF, 100 ng/mL N-plate and 10 ng/mL of G-CSF, IL-3 and IL-6. For growth curves, transduced CB CD34^+^ cells were expanded on the co-culture system with MS5-stromal cells for 35 days. For colony formation assays (performed at day 35 of the culture expansion), a total of 300 CB CD34^+^ cells were seeded on methylcellulose (H4230, Stem Cell Technologies, Vancouver, Canada) supplemented with SCF, FLT3-Ligand, N-plate (all 100 ng/mL), and EPO, IL-3, and IL-6 (all 20 ng/mL). After 8 days for CFU-E/BFU-E and 14 days for CFU-G/GM, colonies were identified and counted. All cell cultures were grown at 37°C and 5% CO_2_.

#### Assessment of total and lipid ROS production

Primary AML blasts and AML cell lines were grown for 24 h in standard culture medium and treated with GFC, VEN, 5′Aza and the combinations (as described in the figure legends). Cells were counted, 100,000 removed to FACs tubes, washed with PBS EDTA and spun for 5 min at 450 rcf. Cells were resuspended in PBS EDTA containing probes for total cell CellROX DeepRed (5 μM) (ThermoScientific; C10422) or the lipid ROS probe BODIPY C11 (1.5 μM) (ThermoScientific; D3861). Cells were stained for 30 min at 37°C, washed twice with PBS EDTA and resuspended in 200 μL of PBS ETDA and held on ice for analysis using the BD LSR-II cytometer and analyzed with FlowJo v10.7 software. Cells were grown overnight in 1 mM and 10 mM L-buthionine-sulfoximine (BSO) (Merck; #B2515) for positive controls of total cellular ROS, while RSL3 and ML210 (Medchemexpress) were added to cells for 12 h at 37°C for a positive control of lipid ROS generation.

#### Connectivity map analysis

Using the Connectivity Map (cMAP - https://clue.io/query) we investigated the relationship between key gene sets associated with *ΔNp73*-overexpression and *TP53*mut-like AMLs, and the cataloged response to clinically approved compounds across various cancer cell lines. Specifically, we analyzed genes from four biologically significant gene sets —Reactome activation of gene expression by SREBF/SREBP, Halmos CEBPA targets up, KEGG unsaturated fatty acid beta oxidation, and our generated TP53 AML signature —using cMAP extensive repository of gene expression signatures derived from small molecule treatments, gene overexpression, and knockout experiments. The analysis employed normalized connection scores (NCS), ranked to generate Tau (τ) scores ranging from −100 (reversed phenotype) to 100 (mimicked phenotype). Scores of ±90 or higher were considered as significantly strong for downstream validations. Our input included genes upregulated in ΔNp73-overexpressing cells and significantly elevated in patients with *TP53*mut-like AMLs/ΔNp73^high^. Using cMAP’s *reverse mode* configuration, we identified small molecules capable of reversing the input gene signatures. The query generated four ranked lists of drugs with τ scores spanning −100 to 100. Low τ scores identified compounds capable of counteracting the expression patterns associated with ΔNp73 overexpression and *TP53*mut-like AMLs. Median statistical analysis of the results consistently identified the adrenergic receptor-related drug guanfacine, which scored below −90 in all lists. This finding suggests that guanfacine effectively downregulates the genetic programs linked to ΔNp73 overexpression and *TP53*mut-like AML phenotypes.

### Quantification and statistical analysis

Survival analyzes were performed in AML patients treated with intensive chemotherapy (3 + 7 scheme) as an induction protocol. Overall survival (OS) was defined as the time from diagnosis to death from any cause related to the disease, those alive or lost to follow-up were censored at the date last known alive. Univariate and multivariate proportional hazards regression analysis was performed for potential prognostic factors for OS. Potential prognostic factors examined and included in multivariable regression analysis were European Leukemia Net 2022 risk stratification, age at diagnosis (analyzed as continuous variable), gender, and the *TP53* mutational status (as a categorical variable: *TP53*wt versus *TP53*mut-like versus *TP53*mut) or the *ΔNp73* expression (as continuous and categorical variables – high versus low). The proportional hazards (PH) assumption for each continuous variable of interest was tested. Linearity assumption for all continuous variables was examined in logistic and PH models using restricted cubic spline estimates of the relationship between the continuous variable and log relative hazard/risk. Descriptive analyses were performed for patient baseline features. Fisher’s exact test or Chi-square test, as appropriate, was used to compare categorical variables. Mann-Whitney or Kruskal-Wallis test was used to compare continuous variables. Details of the statistical analysis and clinical endpoints were described elsewhere. All *p* values were two sided with a significance level of 0.05. All statistical analyses were performed using the statistical package for the social sciences (SPSS) 19.0 and R 3.3.2 (The CRAN project, www.r-project.org) software. Graphs were performed using GraphPad Prism 9 (GraphPad Software, Boston, USA). Statistical tests are also specified in each figure legend, and only relevant comparisons were plotted.

## Supplemental information

Document S1. Figures S1–S6

Table S1. Gene sets associated with TP53mut and TP53mut-like/high ΔNp73-expressing AML samples/models List of gene sets used for Figures 1B, 1J, 2D, and 3F.

Table S2. List of genes that comprehend the TP53 AML signature generated in this manuscript List of 157 differentially expressed in TP53mut AML cases identified in the analysis described in Figure 1A.

Table S3. Patient classification for the multiple AML datasets regarding the TP53mut-like status Patients included in the TCGA, HOVON (GSE6891) and BeatAML cohorts were classified according to the TP53 AML signature and classified as wild-type (WT), TP53mut (Mut) and TP53mut-like (Mut_like).

Table S4. RNA-seq counts for MOLM13 empty vector, ΔNp73-overexpression, and TP73 + 24 kb knockout cells Normalized counts for the biological replicates of the edited MOLM13 cells included in the study. EV, empty vector; OE, ΔNp73-overexpression and KO, TP73 + 24kb knockout cells.

Table S5. ChIP-seq MACS2 peak calling for promoter regions in GFP-ΔNp73-overexpression cells Annotated peak calling analysis of ΔNp73 bound regions in the MOLM13 ΔNp73-overexpressing cells, using an annotation spanning a +-5kb distance from the transcription start site (TSS).

Table S6. List of primers and guide RNAs used throughout the manuscript List of primers used for gene expression analysis, ChIP-qPCR analysis and guide RNAs used to generate the MOLM13 knockout model for the *TP73* + 24kb cells.

Document S2. Article plus supplemental information
