# Supplementary material for: ΔNp73 isoform defines a TP53-mutant-like poor-risk subgroup of acute myeloid leukemia
Source: Cell Rep Med. 2026 Jan 8;7(1):102540. doi: 10.1016/j.xcrm.2025.102540 (PMC12866144; doi:10.1016/j.xcrm.2025.102540)
Supplement: Document S1. Figures S1–S6 [file mmc1.pdf]

Cell Reports Medicine, Volume *7*

Supplemental information

ΔNp73 isoform defines a *TP53*-mutant-like poor-risk subgroup of acute myeloid leukemia

Diego A. Pereira-Martins, Cesar Ortiz, Isabel Weinhäuser, Albertus T.J. Wierenga, Vincent van den Boom, Fatemeh Mojallali, Dominique Sternadt, Nisha K. van der Meer, Shanna M. Hogeling, Thiago M. Bianco, Prodromos Chatzikyriakou, Douglas R. Silveira, Emanuele Ammatuna, Antonio R. Lucena-Araujo, Lynn Quek, Gerwin Huls, Eduardo M. Rego, and Jan Jacob Schuringa

Supplemental Figures

ΔNp73 **isoform defines a new** ***TP53*****mutant-like poor risk subgroup of acute myeloid leukemia**

Diego A Pereira-Martins[1,2,3*], Cesar Ortiz[2,3], Isabel Weinhäuser[1,2], Albertus T J Wierenga[1], Vincent van den Boom[1], Fatemeh Mojallali[1], Dominique Sternadt[1], Nisha K van der Meer[1], Shanna M Hogeling[1], Thiago M Bianco[2], Prodromos Chatzikyriakou[4], Douglas R Silveira[4], Emanuele Ammatuna[1], Antonio R Lucena-Araujo[5], Lynn Quek[4], Gerwin Huls[1], Eduardo M Rego[2,3] and Jan Jacob Schuringa[1*].

Affiliations:

[1]Department of Hematology, University Medical Center Groningen, University of Groningen, Groningen, the Netherlands;

[2]Department of Medical Imaging, Haematology, and Oncology, Ribeirão Preto Medical School, University of São Paulo, Ribeirão Preto, SP, Brazil; Center for Cell Based Therapy, São Paulo Research Foundation, Ribeirão Preto, SP, Brazil;

[3]Hematology Division, LIM31, Faculdade de Medicina, University of São Paulo, São Paulo, Brazil;

[4]Myeloid Leukaemia Genomics and Biology Group, School of Cancer and Pharmaceutical Sciences, King's College London, London, UK;

[5]Department of Genetics, Federal University of Pernambuco, Recife, Brazil

Supplemental figure legends

Supplemental Figure S1

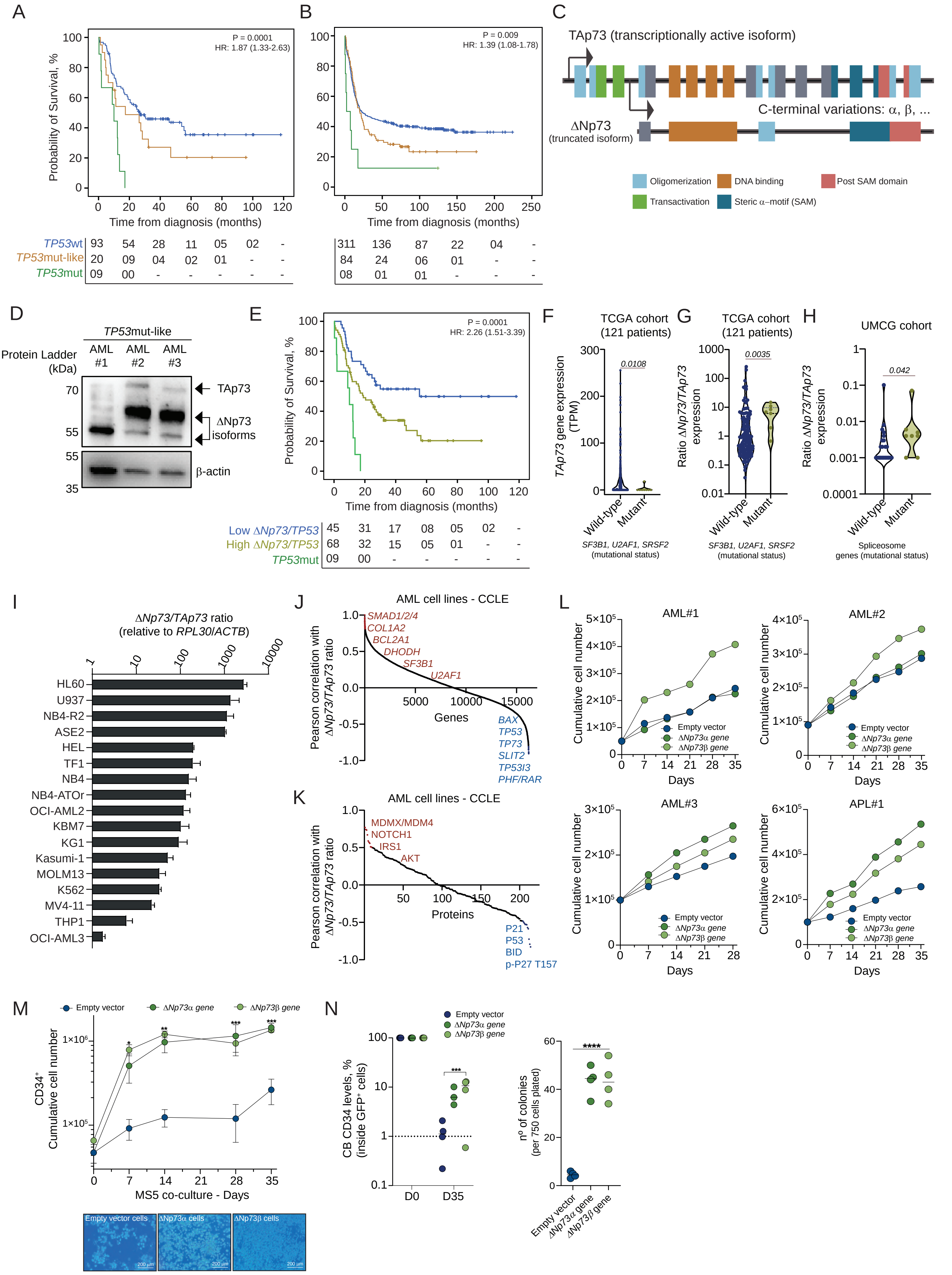

Figure S1. High expression of ΔNp73 is associated with downregulation of TP53 signaling and promotes cell proliferation and survival in hematopoietic cells.

(A-B) Patient survival: The probability of overall survival, OS in AML patients treated with 3+7 based protocols in the TCGA (A) and HOVON (GSE6891) (B) cohorts. Patients were categorized according to the *TP53* mutational status into *TP53*wt, *TP53*mut-like and *TP53*mut. OS curves were estimated using the Kaplan–Meier method, and the log-rank test was used for comparison.

(C) Schematic representation of the *TP73* gene showing the transcriptionally active isoform (*TAp73*) and the truncated variant *ΔNp73*.

(D) Western blot analysis for ΔNp73 in total cell extracts from *TP53*mut-like CD3-depleted primary AML blasts. Membranes were reprobed with βactin antibodies.

(E) Patient survival: The probability of overall survival, OS in AML patients treated with 3+7 based protocols in the TCGA cohort dichotomized according to the *ΔNp73/TP53* gene expression ratio (high versus low), compared to *TP53*mut patients.

(F-H) Violin plots displaying the expression of *TAp73* (E) and the ratio of expression between ratio of *ΔNp73*/*TAp73* in the TCGA cohort (F) and in the UMCG cohort (n=33) (G), according to the mutational status (mutant vs wild-type) of the spliceosome genes (*SF3B1*, *SRSF2* and *U2AF1*).

(I) The relative quantification of the ratio of expression between the ratio of *ΔNp73*/*TAp73* isoforms (relative to *RPL30*/*ACTB* housekeep genes) in a panel of AML cell lines (n=17 lines). Cell lines are described and indicated in the graph.

(J-K) Hockey stick plots displaying the Pearson correlations between the ratio of *ΔNp73*/*TAp73* and the whole transcriptome (J) and the proteome (K) of the AML cell lines, using the data retrieved from the CCLE dataset[14,15].

(L-M) Cumulative cell count of transduced primary AML cells (ΔNp73-OE isoforms/Empty vector control) (L) and cord-blood (CB) derived $CD34^+$ cells (M) cultured on MS5 for 35 days. Plots display the mean ± standard error of the mean (SEM). Representative pictures from the culture conditions for CB experiments are displayed on the bottom of the panel (M) (n=4). Scale bars (200 μm) are displayed in the lower right corner

(N) Dot plot displaying the $CD34^+$ levels (%, measured by flow cytometry on days 0 and 35 in culture) and the levels number of colonies generated from the $CD34^+$ transduced cells and plated after 21 days in culture. Colonies were scored after 14 days (n=4).

The p-values are indicated in the graphs; $^*p < 0.05$; $^{**}p < 0.01$; $^{***}p < 0.001$, ANOVA and Bonferroni post-test.

Supplemental Figure S2

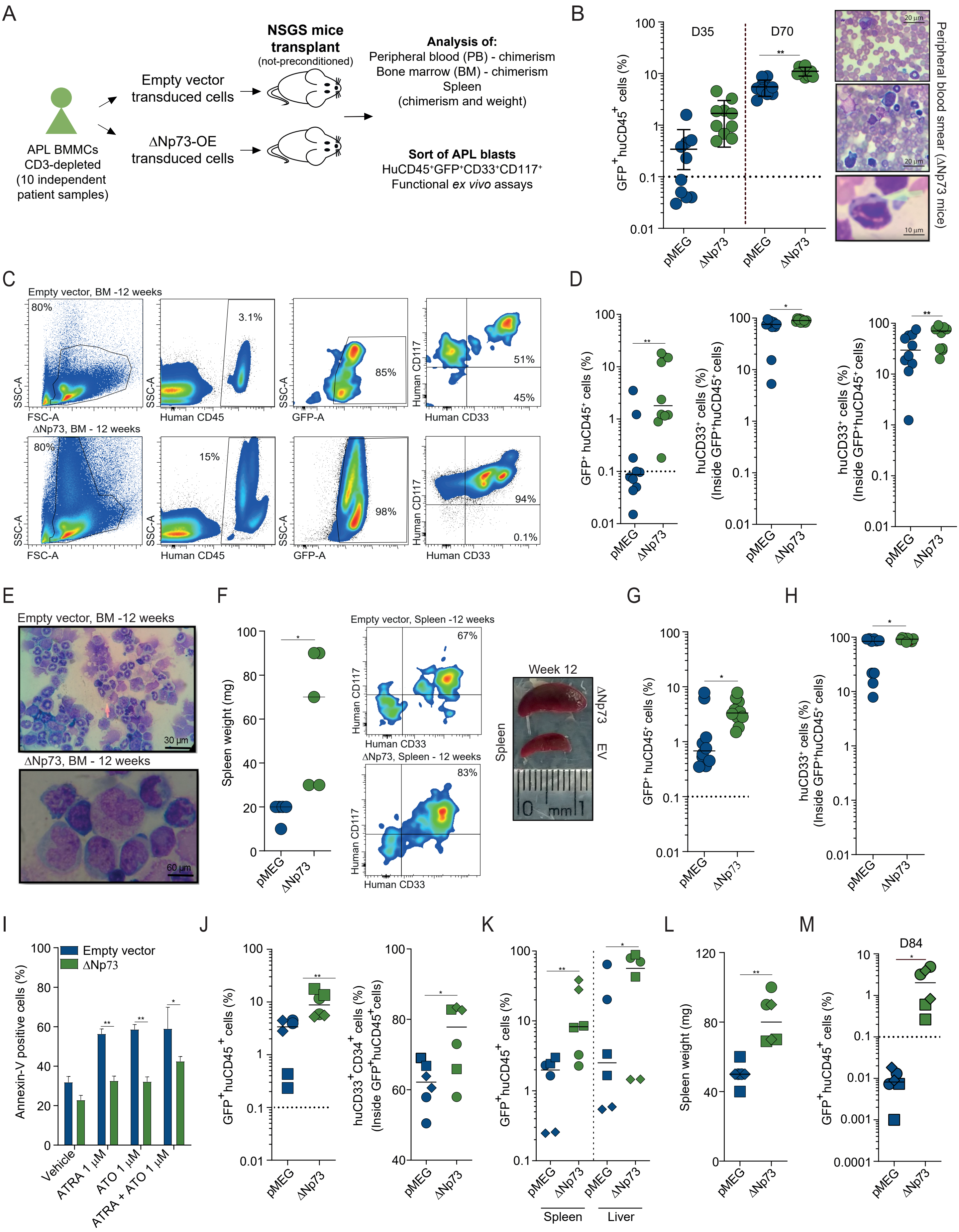

Figure S2. Primary ΔNp73 transduced APL blasts (GFP$^+$ cells) exhibited increased in vivo engraftment in NSGS mice.

(A) Overview of the mouse xenograft for APL. Schematic representation of the generation of the xenograft mouse model for APL engraftment using NSGS mice (n=10).

(B) Scatter plots showing engraftment of donor human GFP$^+$CD45$^+$ cells in the peripheral blood of transplanted mice at day 35 and day 70. Scale bars (20 µm for upper and middle panels and 10 µm for lower panel) are displayed on the lower right corner.

(C) Representative FACS phenotype from a primary murine bone marrow transplanted with human transduced APL blasts with the empty vector (upper panels) or the *ΔNp73* gene (lower panel) at sacrifice (week 12). APL blasts and mature myeloid committed cells were analyzed by flow cytometry using markers against CD117, CD33 and CD11b as indicated (inside the population huCD45$^+$ and GFP$^+$).

(D-E) Scatter plots show engraftment of donor human CD45$^+$ cells (inside the GFP$^+$ population, left panel), and human GFP$^+$CD33$^+$ cells in the peripheral blood (middle panel) and bone marrow (right panel). (E) Representative images of May-Grünwald-Giemsa-stained bone marrow smears of engrafted primary APL blasts (Empty vector and ΔNp73-OE) at sacrifice. Scale bars (upper panel: 30 µm and lower panel: 60 µm) are displayed in the lower right corner.

(F-H) Spleen weight (F) with representative FACS phenotype of engrafted cells and levels of GFP$^+$huCD45$^+$ (G) and huCD33$^+$ (H) at sacrifice. Data were expressed as median values.

(I) *Ex vivo* analysis of transduced APL blasts reinforces *in vitro* findings. Incubation of bone marrow sorted APL blasts cells (GFP$^+$CD45$^+$CD117$^+$CD33$^+$) from pMEG/ΔNp73 engrafted mice, with ATRA, ATO and the combination (1 µM each) led to reduced drug-induced apoptosis over the course of 72 hours in ΔNp73 cells (n=3).

(J-M) Primary AML samples were transduced with control (pMEG) or ΔNp73-OE lentivectors and injected into MISTRG mice. Three independent patient samples were used indicated by different symbols, two mice were injected per patient group. Mice were sacrificed at week 12 after which human chimerism in the BM (L, percentage human

CD45, left panel, and percentage human $CD34^+/CD33^+$ within the human $CD45^+$ population, right panel) was determined. Human chimerism was also determined in the spleen and liver (K) and spleen weight is also shown (L).

Data were expressed as mean ± standard error of the mean. The p-values and cell lines are indicated in the graphs; $*p < 0.05$; $**p < 0.01$; $***p < 0.001$, ANOVA and Bonferroni post-test.

Supplemental Figure S3

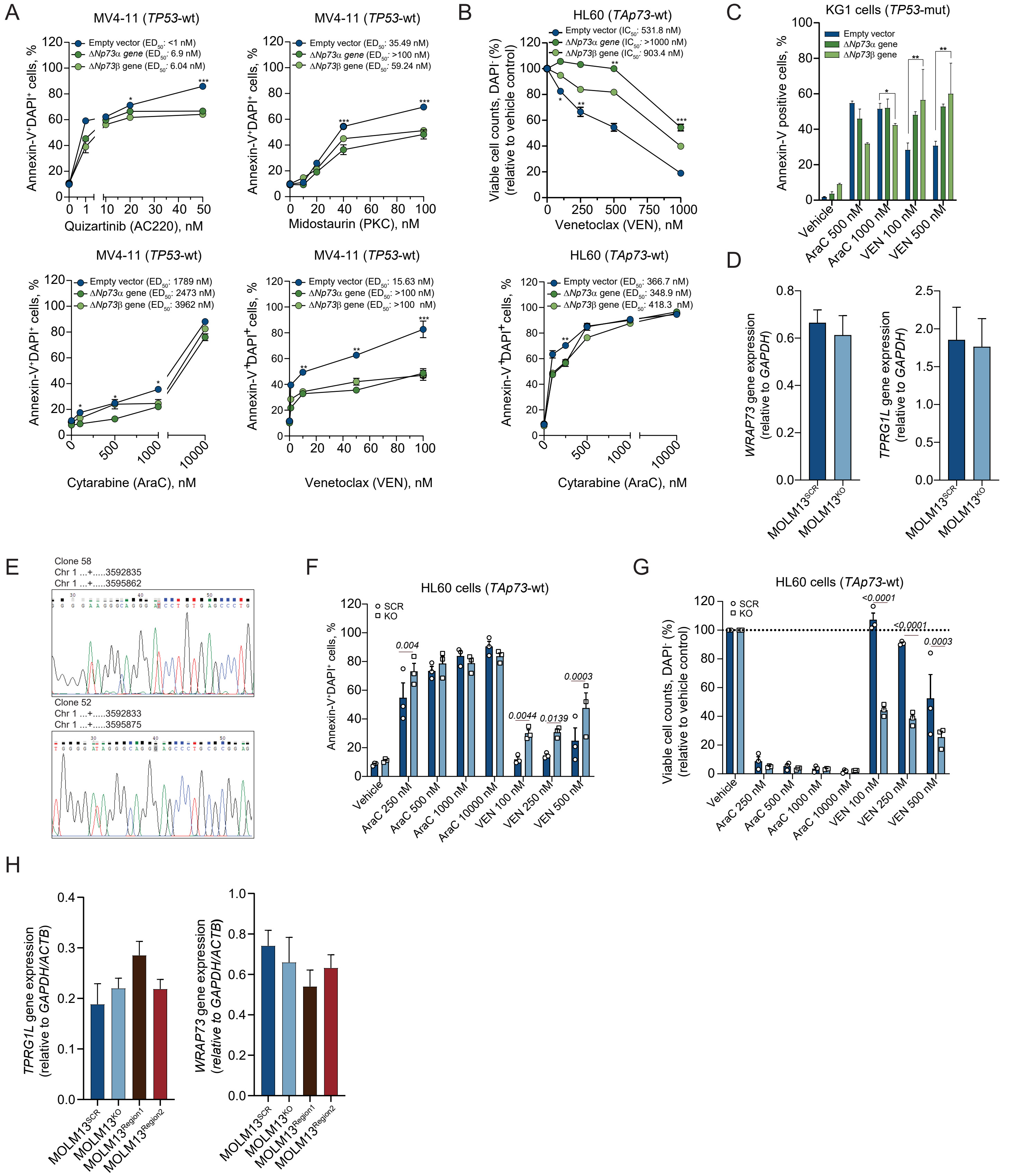

Figure S3. ΔNp73 expression is associated with drug resistance in a TP53wt context.

(A-B) MV4-11 cells (ΔNp73-OE and empty vector control) were treated with FLT3-inhibitors quizartinib (AC220), midostaurin (PKC) and AML-related drugs venetoclax (VEN) and cytarabine (AraC) (A) and HL60 cells were treated with VEN and AraC (B) for 72 hours. Apoptosis and viable cell numbers were assessed by flow cytometry. Experiments were performed in quadruplicates. Results are expressed as the mean ± standard error of the mean (SEM). ED_{50}: half maximal effective concentration (n=4).

(C) Drug-induced apoptosis in KG1 cells (ΔNp73-OE and empty vector control) treated with AML-related drugs (AraC and VEN, concentrations indicated in the plots - 72 h) detected by flow cytometry (n=4).

(D) Relative mRNA expression levels of the *TP73* neighboring genes (*TPRG1L* and *WRAP73*) after CRISPR-Cas9-mediated intragenic enhancer excision in MOLM13 cells (subsequently referred to as MOLM13-KO cells) (n=4).

(E) Targeted Sanger sequencing of TP73 intragenic enhancer region in MOLM13 KO cells. Annotation on the top shows the chromosomal coordinates for the initial point prior to the cut, and second annotation shows the point for the first base after the cut. For clones 58 and 52, we can see a removal of 3,042 bp.

(F-G) Drug-induced apoptosis (F) and viable cell counts (G) in HL60-KO cells treated with AML-related drugs (drugs and concentrations indicated in the plots - 72 h) detected by flow cytometry.

(H) Relative mRNA expression levels of the *TP73* neighboring genes (*TPRG1L* and *WRAP73*) after CRISPR-Cas9-mediated intragenic enhancer excision of the separate regions 1 and 2 of the intragenic enhancer promoter of the *TP73* gene in MOLM13 cells (n=4).

The p-values and cell lines are indicated in the graphs; *$p < 0.05$; **$p < 0.01$; ***$p < 0.001$, ANOVA and Bonferroni post-test.

Supplemental Figure S4

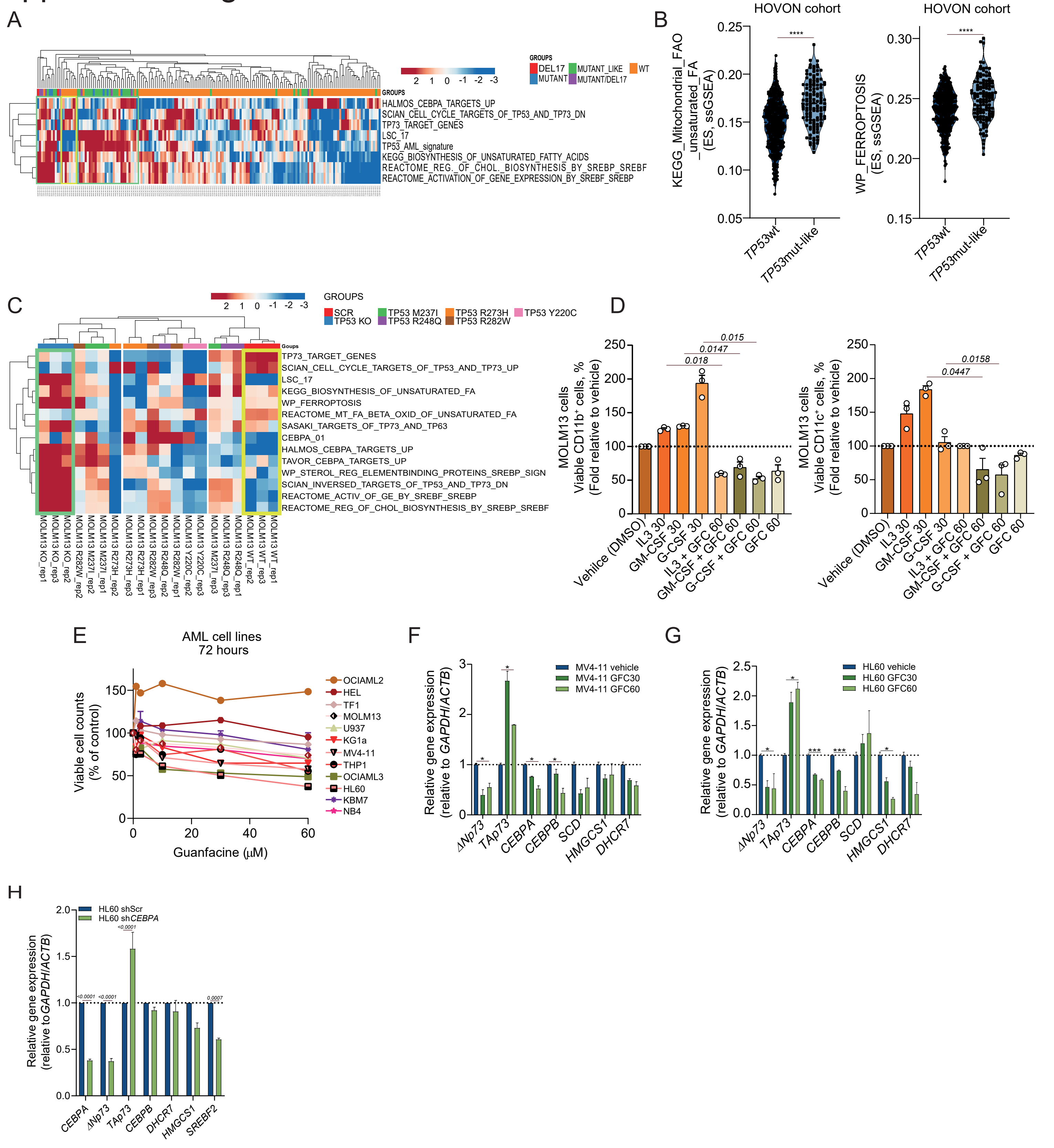

Figure S4. Alterations in *TP53* gene are associated with deregulation of the CEBPA downstream signaling in AML.

(A) Heatmap representation of the ES for ssGSEA processes associated with *TP53*mut-like AMLs in AML patients included in the TCGA cohort (n=173). Patients were categorized as *TP53*wt (with and without deletion of the *TP53* gene locus on chromosome 17), *TP53*mut (with and without deletion of the *TP53* gene locus on chromosome 17), and *TP53*mut-like.

(B) Violin plots displaying the ES for the KEGG_Mitochondrial_FAO_unsaturated_FA and the WP_Ferroptosis signature for AML patients included in the HOVON cohort (n=517). Patients were categorized according to the *TP53* mutational status into *TP53*wt and *TP53*mut-like.

(C) Heatmap representation of the ES for ssGSEA processes depicted in panel (A) in a cohort of MOLM13 cells with different *TP53* mutations and KO[6].

(D) Bar plots displaying the flow cytometry analysis of the $CD11b^+$ and $CD11c^+$ cells in MOLM13 cells treated with different hematopoietic-related cytokines (IL-3, G-CSF and GM-CSF, 30 ng/mL) in the presence or absence of guanfacine (GFC, 30 and 60 µM) for 72 hours.

(E) Dose-response cytotoxicity was analyzed using an Annexin-V/DAPI staining method in a panel of AML cell lines treated with vehicle or increasing concentrations of GFC for 72 h. Values are expressed as the percentage of viable cells for each condition relative to vehicle-treated cells (n=4).

(F) Relative mRNA expression levels of *TP73* isoforms and *CEBPA/CEBPB* and its related targets at baseline and upon guanfacine (GFC) treatment (30 and 60 µM) in MV4-11 cells (48 hours) (n=4).

(G-H) Relative mRNA expression levels of the same targets as described in panel (F) in HL60 cells treated with GFC (30 and 60 µM, 48 hours, G) transduced with shRNA targeting the *CEBPA* gene and the scrambled control (H) (n=4).

The p-values and cell lines are indicated in the graphs; *$p < 0.05$; **$p < 0.01$; ***$p < 0.001$, ANOVA and Bonferroni post-test.

Supplemental Figure S5

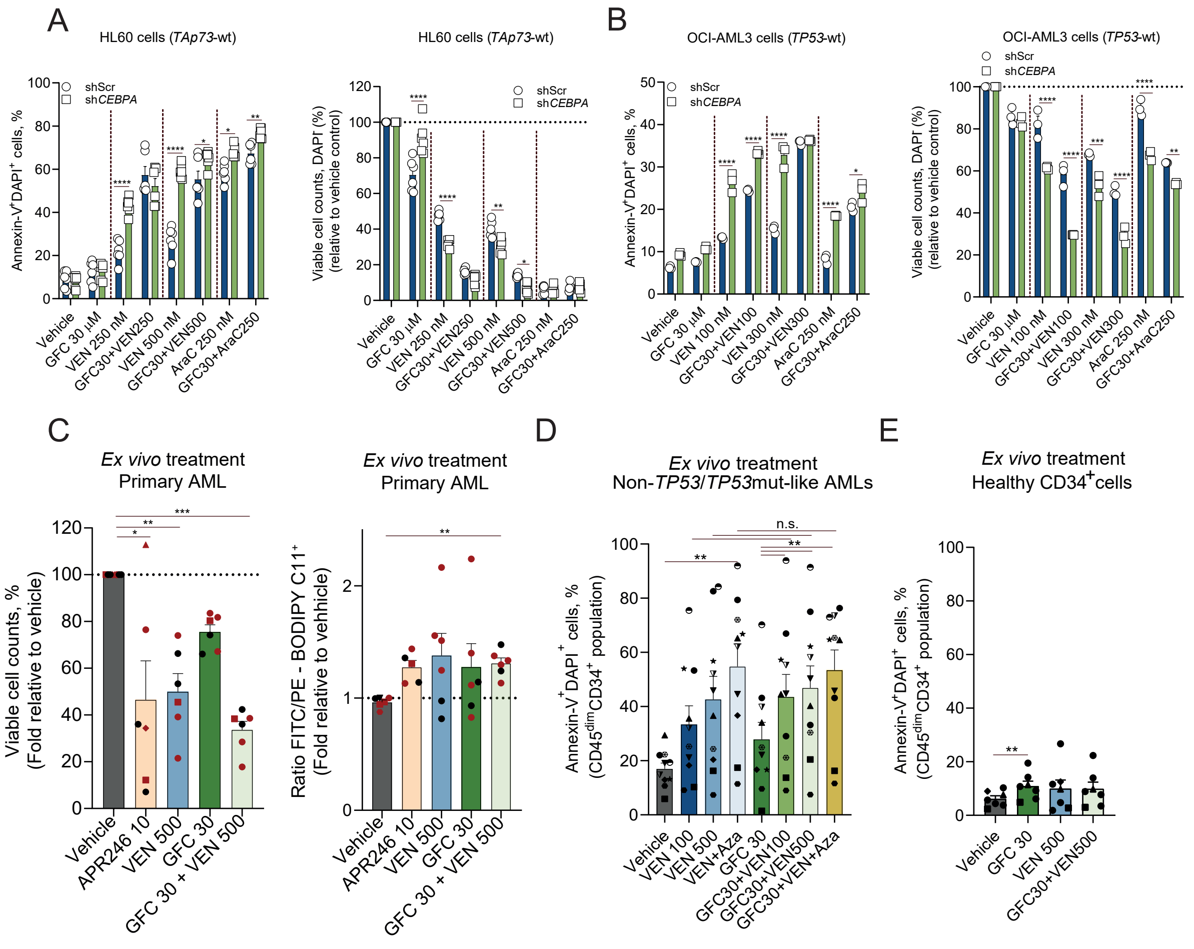

Figure S5. Pharmacological and genetic inhibition of CEBPA potentializes cytotoxic therapy in AML.

(A-B) Drug-induced apoptosis and viable cell counts in HL60 (n=6) (A) and OCI-AML3 (n=3) (B) shCEBPA/shScr cells treated with VEN and AraC alone or in combination with GFC (concentrations indicated in the plots - 72 h) detected by flow cytometry.

(C) Viable cell counts (left plot) and ratio FITC/PE of the BODIPY C11^{TM} probe (right panel) detected by flow cytometry in gated human CD45^{dim}CD34^{+} (or CD117^{+} cells for CD34^{-} AMLs) of *ex vivo* treated AML samples categorized as *TP53*mut (red dots) and *TP53*mut-like (black dots). Values were normalized by vehicle controls (n=6).

(D) Apoptosis was detected by flow cytometry in gated human CD45dimCD34^{+} (or CD117^{+} cells for CD34^{-} AMLs) of *ex vivo* treated AML samples categorized as non-*TP53*mut/mut-like (including *CEBPA* mutant AMLs, n=10). Cells were treated with vehicle, VEN (100 and 500 nM), VEN+Aza (VEN 100 nM + 5`Aza 1.5 μM), in the presence or absence of GFC (30 μM) for 72 h. APR-246, eprenetapopt.

(E) As in (D) but now healthy CD34^{+} cells isolated from old bone marrow samples (n=7) were used.

Bar graphs represent the mean ± SEM of all the independent patients screened, each point represents a patient. The p-values and cell lines are indicated in the graphs; *p < 0.05; **p < 0.01; ***p < 0.001, ANOVA and Bonferroni post-test.

Supplemental Figure S6

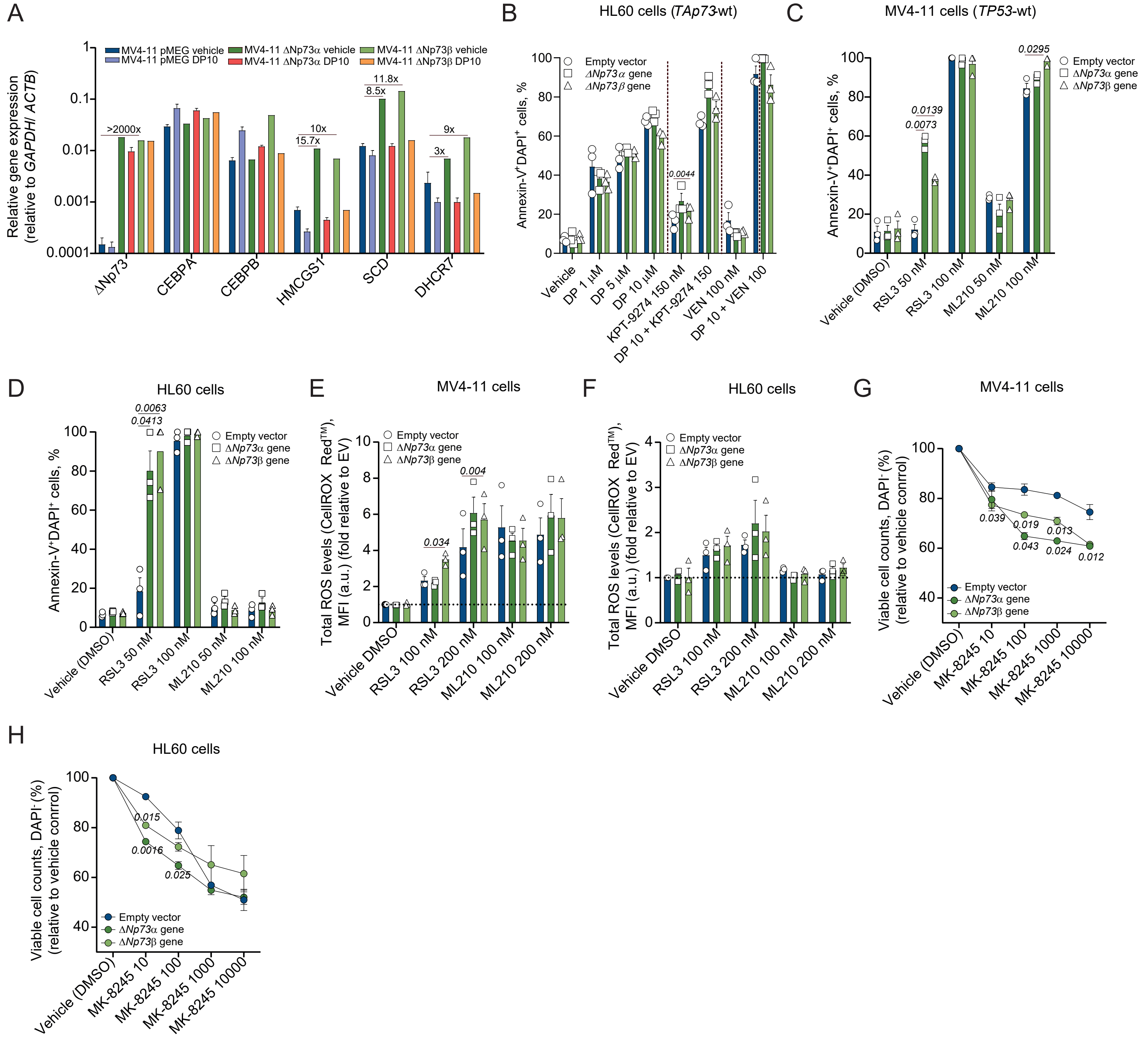

Figure S6. SREBP inhibition overcomes the ΔNp73-induced drug resistance in AML. (A) Relative mRNA expression levels of Δ*Np73*, *CEBPA/CEBPB* and its related targets at baseline and upon dipyridamole (DP) treatment (10 μM) in MV4-11 ΔNp73-OE/empty vector (pMEG) cells (n=4).

(B) Drug-induced apoptosis in HL60 cells (ΔNp73-OE and empty vector control) treated with ferroptosis-related drugs KPT-9274 (NAMPT inhibitor[20]) and DP alone or in combination with VEN (concentrations indicated in the plots - 72 h) detected by flow cytometry (n=3).

(C-F) Drug-induced apoptosis in MV4-11 (C), HL60 cells (D) and total ROS levels in MV4-11 (E) and HL60 cells (F) (ΔNp73-OE and empty vector control) treated with the GPX4 inhibitors RSL3 and ML210[22] (concentrations indicated in the plots - 72 h) detected by flow cytometry (n=3).

(G-H) Viable cell counts of MV4-11 (G) and HL60 (H) cells (ΔNp73-OE and empty vector control) treated with the SCD-inhibitor MK-8245 (concentrations indicated in the plots - 72 h) detected by flow cytometry (n=4).

The p-values and cell types are indicated in the graphs; $*p < 0.05$; $**p < 0.01$; $***p < 0.001$, ANOVA and Bonferroni post-test.
